# Determination of design requirements and characteristic analysis of powertrain configurations for electric tractors based on actual agricultural workload

**DOI:** 10.1038/s41598-026-44453-0

**Published:** 2026-03-21

**Authors:** Da-Vin Ahn, Ji-Tae Kim, Kyeongdae Kim, Gyu-Ha Han, Seung-Je Cho, Wongun Kim, Young-Jun Park

**Affiliations:** 1 Jeonbuk Technology Application Division (Specialized Machinery) Specialized Machinery and Robotics Group, Korea Institute of Industrial Technology, Gimje, 54325 South Korea; 2 Department of Convergence Biosystems Engineering, Chonnam National University, 77 Yongbong-ro, Buk-gu, Gwangju, 61186 South Korea; 3 Department of Biosystems Engineering, Seoul National University, Seoul, 08826 South Korea; 4 Convergence Major in Global Smart Farm, Seoul National University, Seoul, 08826 South Korea; 5 Research Institute of Agriculture and Life Science, Seoul National University, Seoul, 08826 South Korea

**Keywords:** Electric tractor, tillage operations, system requirements, powertrain design, Energy science and technology, Engineering

## Abstract

Determining the design requirements for an electric tractor is crucial for its optimal configuration, as specifications vary across different agricultural operations, field sizes, and field characteristics. This study delineates the design requirements for electric tractors based on agricultural workload assessments. Subsequently, electric-tractor powertrain configurations were proposed, and their performance characteristics were analyzed. Initially, the agricultural workload for tractors with internal combustion engines of comparable class was measured during plow tillage, rotary tillage, and driving operations. Following this, the design requirements for electric tractors were conceptualized as power envelope curves derived from the measured agricultural workloads. These requirements were categorized into traction power and PTO (power take-off) power design requirements, considering the distinctive features of tractor operations in agriculture. Based on these stipulated requirements, three electric-tractor powertrain configurations were suggested. The performance of these configurations was evaluated concerning motor specifications, structural complexity, powertrain efficiency, and control difficulty. The results can be used as a basis for a methodology for establishing electric-tractor design requirements and for devising powertrain configurations, serving as a significant resource for future electric-tractor design and development.

**Supplementary Information:**

The online version contains supplementary material available at 10.1038/s41598-026-44453-0.

## Introduction

An agricultural tractor is a machine designed to provide mechanical power for various farming tasks, including towing implements such as plows and trailers, supplying rotational power to driven machinery such as rotavators and mowers, and providing hydraulic power for front loaders. Traditionally powered by diesel engines, agricultural tractors must offer a wide torque and speed range to accommodate diverse agricultural operations under varying load conditions. For instance, plowing requires high torque at low speeds, driving operations necessitate low torque at high speeds, and rotary tillage involves transferring engine power to a power take-off (PTO) shaft. Historically, tractors have relied on internal combustion engines (ICEs) linked to transmissions with complex structures and multiple gear ratios to meet the varied demands of agricultural work. However, this configuration presents significant inefficiencies, including considerable power loss across the numerous transmission ratios^1^. Furthermore, the reliance on ICEs introduces challenges related to low energy efficiency, high fuel consumption, and increased emissions^2–4^. The recent implementation of the Stage V standard in Europe, which imposes stricter limits on particulate matter and introduces an ammonia emissions standard, has prompted several countries, including Korea, to adopt similar regulations^5^. Consequently, there is an urgent need within the agricultural machinery sector to enhance energy efficiency and develop alternatives that reduce or eliminate emissions and pollution^6^. The electrification of agricultural machinery, with electric tractors as environmentally friendly options, has been proposed as a solution^7^.

Research on the electrification of tractors primarily focuses on two areas: the design of powertrain configurations for electric tractors^8–11^ and the development of appropriate control algorithms for these powertrains to minimize energy consumption under various agricultural conditions, including plow tillage, rotary tillage, and driving^6,12,13^. The specifications for tractors depend on the type of farming operation, field size, and field characteristics^14^. Therefore, it is critical to establish the design requirements for tractors before advancing research on powertrain structures and controls in the development of electric tractors.

The design requirements for an electric tractor are determined by two critical considerations: (1) the electric tractor’s ability to perform farming tasks at par with a target-specification ICE tractor, and (2) the tractor’s capability to meet the required workload conditions, including torque and speed specifications. If the goal is for the electric tractor to deliver power equivalent to an ICE tractor of a given specification, the engine power of the ICE tractor can be selected as the benchmark for the electric tractor’s design requirements. However, the necessity for an ICE tractor’s engine to continuously operate the hydraulic system, coupled with the efficiency losses from a complex, multistage transmission^15^, suggests that basing the electric tractor’s design solely on engine power could lead to overdesign. Such an approach is not optimal, as it equips the electric tractor with a drive motor and transmission capacity exceeding the actual needs. Thus, identifying electric-tractor design requirements necessitates an assessment of the workload demanded by various agricultural operations, using the target specification of an ICE tractor as a reference.

Cho et al. implemented measurement sensors and data acquisition systems on an 80 kW tractor to measure the load required for each type of agricultural activity. Workload data included measurements of traction force, travel speed, PTO torque, and rotational speed. During rotary operations, engine power reached 40 kW, with PTO power averaging 83.8% ^16^. Ryu et al. measured the power transmission efficiency of tractor transmissions during driving operations using a 30 kW tractor equipped with a four-speed main transmission and a four-speed sub-transmission^17^. The efficiency, calculated as the ratio of input power to output power, was determined by attaching sensors to both the transmission input (input shaft) and output (wheel axle). The results revealed that the driveline’s power transmission efficiency varied between 56% and 86%, averaging 72.5%. Transmission drag torque, responsible for 61% of power losses, emerged as the primary inefficiency factor in multispeed transmissions. This insight highlighted the significance of minimizing gear stages in the electric tractor’s transmission design. Kim et al. assessed the load on an 82 kW tractor during plow tillage operations^18^. Rather than directly measuring engine torque and speed, engine power was indirectly inferred from separate measurements of hydraulic, PTO, and traction system loads, subsequently converted into engine power. The investigation noted an increase in engine torque with higher gear stages during plowing, suggesting that future research might derive optimal transmission gear ratios for plow tillage based on equivalent workloads. However, as the indirectly determined engine power includes loads from the hydraulic system, designing transmissions based solely on this approach could result in overdesign. The aforementioned studies on ‘tractor workload measurement and analysis’ and ‘tractor powertrain analysis’ were primarily aimed at evaluating the workloads on tractors to estimate power consumption and losses or to assess power transmission efficiency. No research that established tractor design requirements based on load data or analyzed the characteristics of tractor powertrain was identified.

This study aims to establish the design requirements for an electric tractor through the measurement and analysis of loads generated during various agricultural operations with an ICE tractor of targeted specifications. Additionally, it focuses on examining the characteristics of the proposed electric-tractor powertrain configurations. Our investigation includes three primary stages: The first stage involves the development of an agricultural workload measurement system and the subsequent measurement of agricultural workloads through operational tests with a target ICE tractor. The farming operations considered include plow tillage and driving operation utilizing traction power, alongside rotary tillage employing both traction power and PTO power. The second stage involves the division of electric-tractor design requirements into traction power and PTO power, based on the collected agricultural workload data. The third stage involves the analysis of the characteristics of three proposed powertrain configurations, informed by the electric-tractor design requirements.

The novelty of this study is threefold: (1) it categorizes tractor agricultural workload data into traction power and PTO power to reflect the distinct nature of tractor work; (2) it introduces a methodology for defining electric-tractor design requirements by integrating agricultural workload data with field characteristics; (3) it proposes and compares representative electric-tractor powertrain configurations based on electric-tractor design requirements.

## Methods

### Test layout

#### Tractor and instrumentation system

For this study, a tractor equipped with a 46 kW EU Stage III engine (TCD 2.9L4, DEUTZ) was utilized. The engine delivers a maximum torque of 262 N‧m at a rotational speed of 1800 rpm and a rated power of 46 kW at 2300 rpm. The tractor features a power shuttle transmission, enabling automatic shifting between forward and reverse. This transmission includes a four-step main shift gear (stages 1–4), a 3-step auxiliary shift gear (L, M, H), and a creep mode. The PTO powertrain system operates at three speeds, with PTO 1st gearat 540 rpm, PTO 2nd gear at 750 rpm, and PTO 3rd- gear at 1000 rpm, all measured at the engine’s rated speed. The tractor’s dimensions are 3,695 mm in length, 1,850 mm in width, and 2,560 mm in height. Table 1 presents the detailed specifications of the tractor used in this study.

**Table 1:** Specifications of tractor used in this study.

Item			Specification
Dimension		Length (mm)	3,695
		Width (mm)	1,850
		Height (mm)	2,560
		Weight (kg)	2,615
		Wheelbase (mm)	2,155
		Minimum ground clearance (mm)	420
Transmission	Main part	Stages	4 stages
	Subpart	Stages	3 stages
	Type	Power shuttle	
Weight distribution		Front (%)	45.9
		Rear (%)	54.1
Tire		Front	11.2–20 8PR
		Rear	14.9–30 8PR
Engine		Type	4 cylinder diesel
		Rated power (kW)	46 @ 2,300 rpm
		Max torque (N‧m)	262 @ 1,800 rpm

Plow tillage was conducted using a six-row moldboard plow (WJSP-6 S, Woongjin Machinery Co. Ltd., Korea). The plow allows for a maximum tillage depth of 200 mm and measures 1,930 mm in length, 1,800 mm in width, and 1,235 mm in height. The specifications of the plow are provided in Table 2.

**Table 2:** Specifications of plow used in this study.

Item		Specification
Type		Moldboard plow
Number of furrows		3
Dimension	Length (mm)	1,930
	Width (mm)	1,800
	Height (mm)	1,235
Required power (kW)		40–52
Weight (kg)		370
Maximum tillage depth (mm)		200

Rotary tillage was performed with a rotavator (WJ185A, Woongjin Machinery Co. Ltd., Korea), capable of a maximum tillage depth of 200 mm. The rotavator’s dimensions are 810 mm × 2,020 mm × 1,130 mm (length × width × height), and its specifications are detailed in Table 3.

**Table 3:** Specifications of rotavator used in this study.

Item		Specification
Dimension	Length (mm)	900
	Width (mm)	2,020
	Height (mm)	1,130
Tillage width (mm)		1,820
Required power (kW)		35.8–41
Weight (kg)		405
Maximum tillage depth (mm)		200
Number of flanges		7
Number of blades		42

#### Sensors and data measurement system

**Fig. 1:**
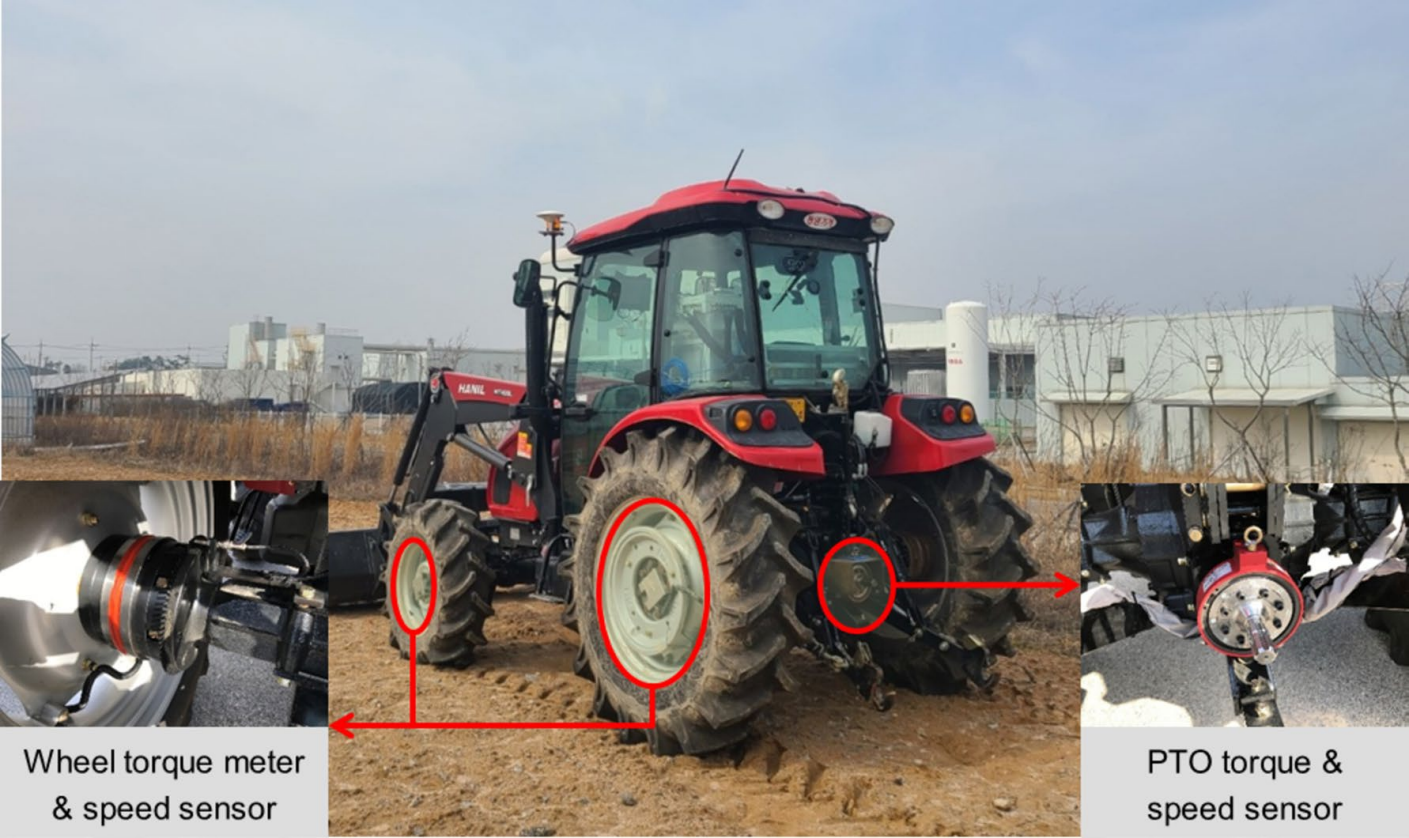
Configuration of the agricultural workload measurement system.

The agricultural workload measurement system was designed to accurately measure wheel torque, wheel speed, PTO torque, and PTO speed as shown in Fig. 1. Torque meters were installed on each of the four wheels (front left, front right, rear left, and rear right), capable of measuring torque up to 25 kN‧m. To calculate the rotational speed of the wheels, gear teeth speed sensors, which detect pulse signals generated by the gear teeth as the wheel shaft rotates, were installed. The PTO torque and speed measurements were facilitated by a combined torque meter and speed sensor mounted on the PTO shaft, with a measurement capacity of up to 5 kN‧m. Table 4 outlines the detailed specifications of the sensors employed in the agricultural workload measurement system. All sensor signals were acquired at a sampling rate of 1000 Hz. Prior to operating-point extraction, the measured signals were filtered using a low-pass filter to reduce high-frequency noise. Outliers were removed by excluding physically implausible values, including samples outside the sensor measurement range and non-physical spikes observed in the wheel-speed signal. Wheel-speed spikes were identified using an objective criterion for isolated abrupt changes and were removed prior to further processing.

**Table 4:** Specifications of sensors used in this study.

Item	Model	Specification
Wheel torque meter	RF-15000-275 / MANNER Sensortelemetrie / Germany	Nominal load: 25 kN‧m Measuring range: −25–25 kN‧m Accuracy: 0.1%
Wheel speed sensor	CYGTS211B / ChenYang Technologies GmbH&Co. KG / Germany	Response frequency: 1–20 kHz Sensing distance: 3 mm
PTO torque & speed sensor	MW_B_5kNm_PCM16 / MANNER Sensortelemetrie / Germany	Nominal load: 5 kN‧m Measuring range: −5–5 kN‧m Maximum speed: 2,000 rpm Accuracy: 0.1%

### Field test

#### Test site and soil property

The field tests for plow and rotary tillage were conducted on a farm located in Seodun-dong, Kwonseon-gu, Suwon-si, Gyeonggi-do, Korea (37°16’09.7’’N, 126°59’26.3’’E), covering an area of 990 m^2^ (18 m × 55 m). Driving operations on an asphalt driveway were performed on a road in Baeksan-myeon Bugari, Kimje-si, Jeollabuk-do (35°51’0.0"N 126°52’12.0"E).

**Fig. 2:**
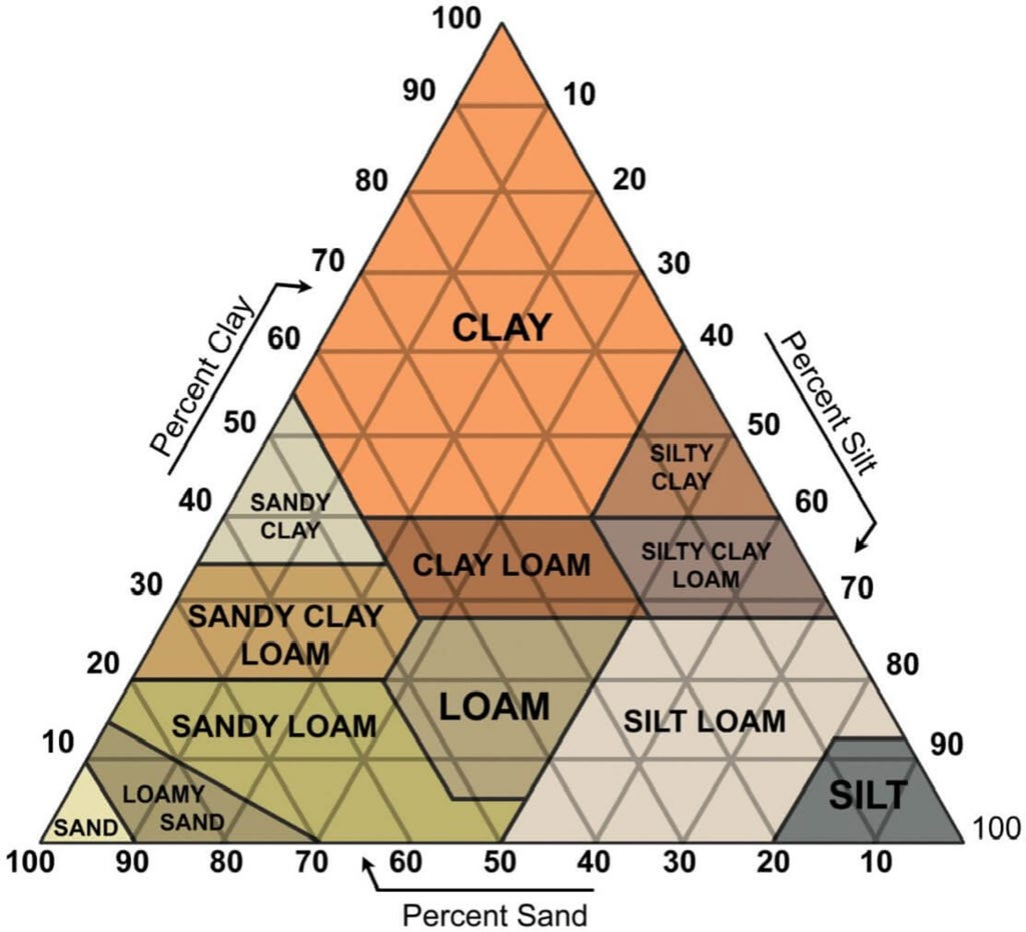
Soil texture diagram of USDA classification.

**Fig. 3:**
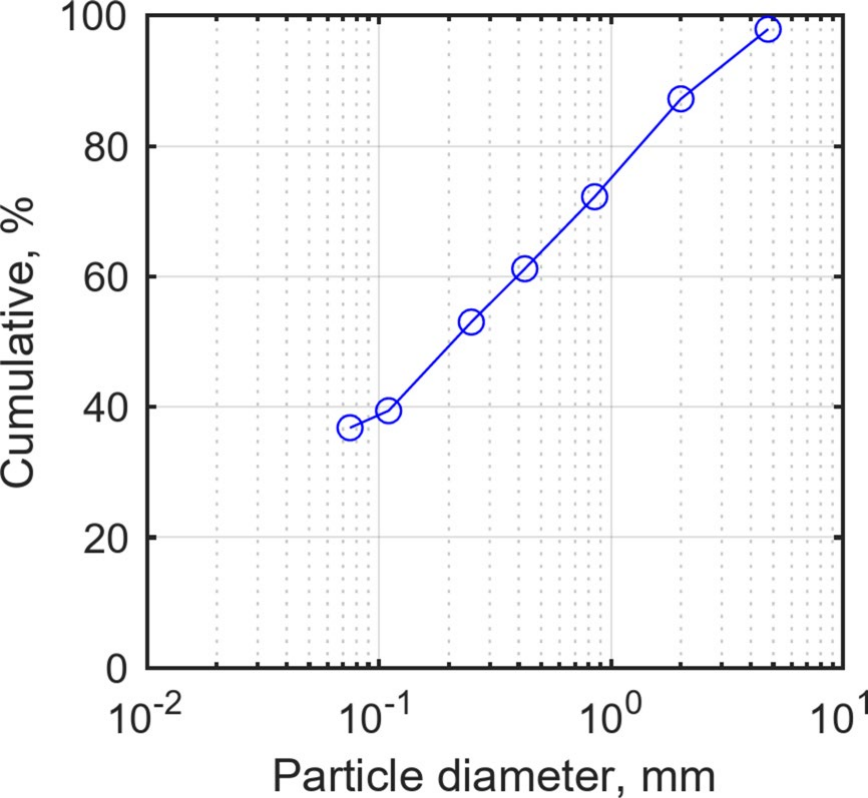
Grain-size distribution curve of test site.

To characterize the soil conditions at the test site prior to the agricultural operation tests, soil texture, bulk density, and cone index were measured. These measurements are reported to describe the test-field conditions and to provide context for the workload measurements. Soil analysis was conducted using sieve analysis and the U.S. Department of Agriculture (USDA) soil classification and was performed at the National Instrumentation Center for Environmental Management at Seoul National University’s College of Agriculture and Life Sciences. According to the USDA soil classification criteria, the soil texture at the test site is sandy loam (Fig. 2). Figure 3 presents the grain-size distribution curve of the test site, and Table 5 lists its soil properties.

**Table 5:** Soil properties of test site.

Soil texture	Soil proportion			Bulk density (g/cm^3^)
	Sand (%)	Silt (%)	Clay (%)	
Sandy loam	58.76	25.12	16.12	1.42

**Fig. 4:**
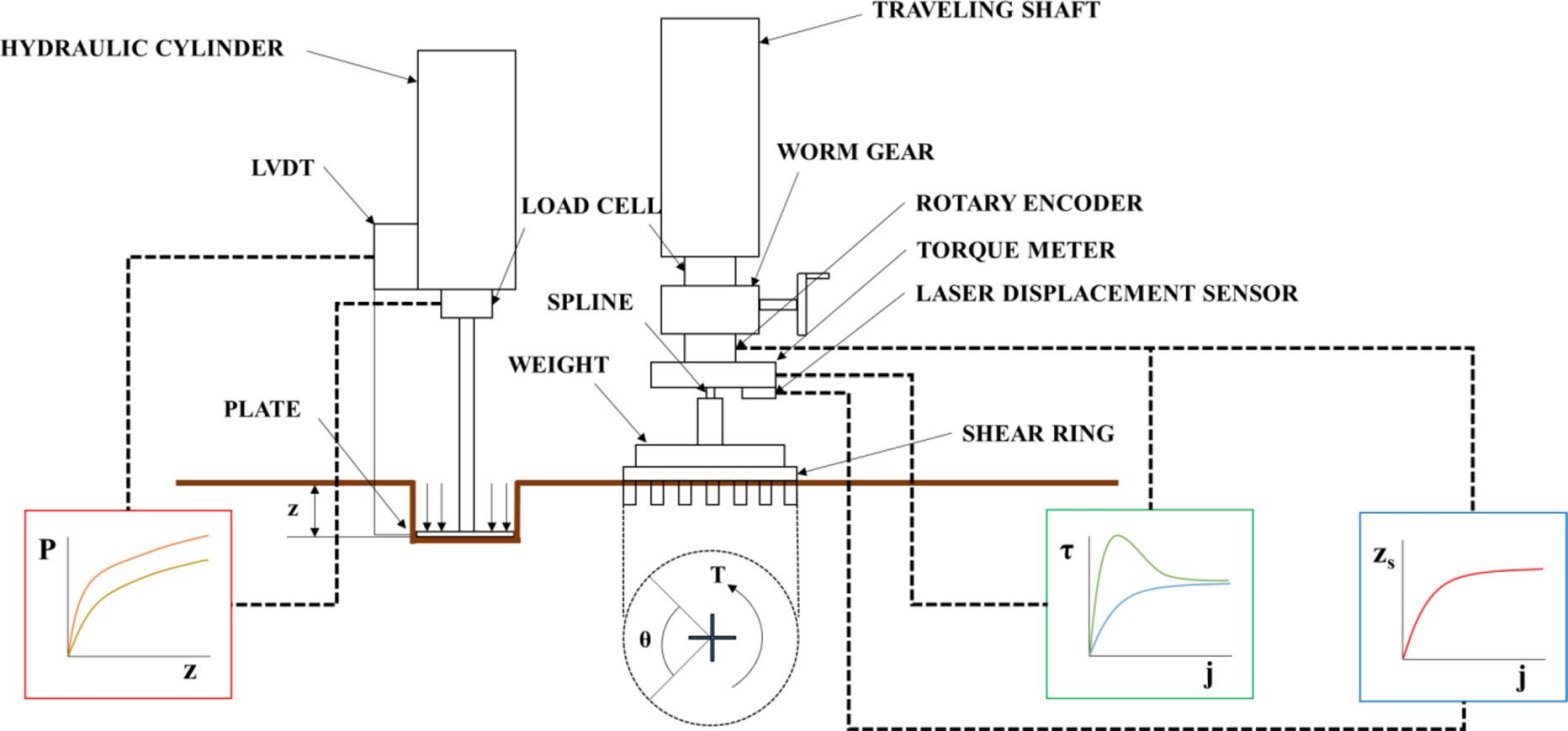
Schematic of bevameter used in this study (Kim et al., 2021).

**Fig. 5:**
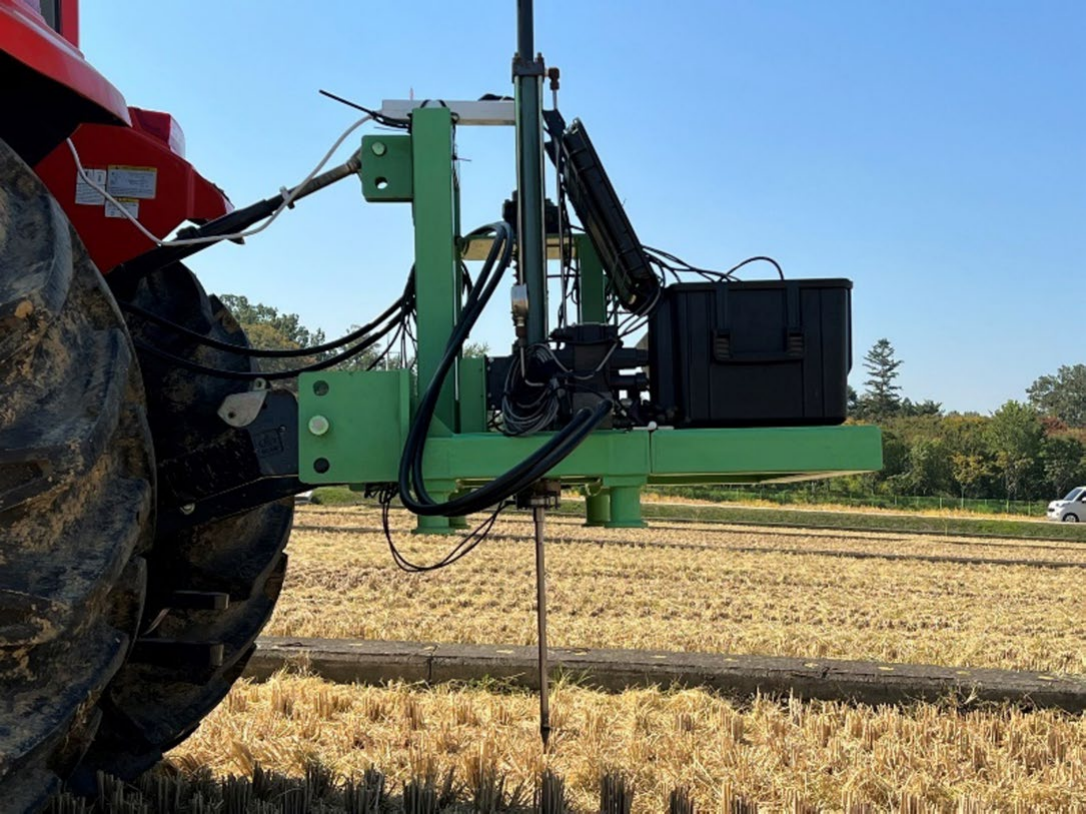
Cone penetrometer attached to bevameter.

**Fig. 6:**
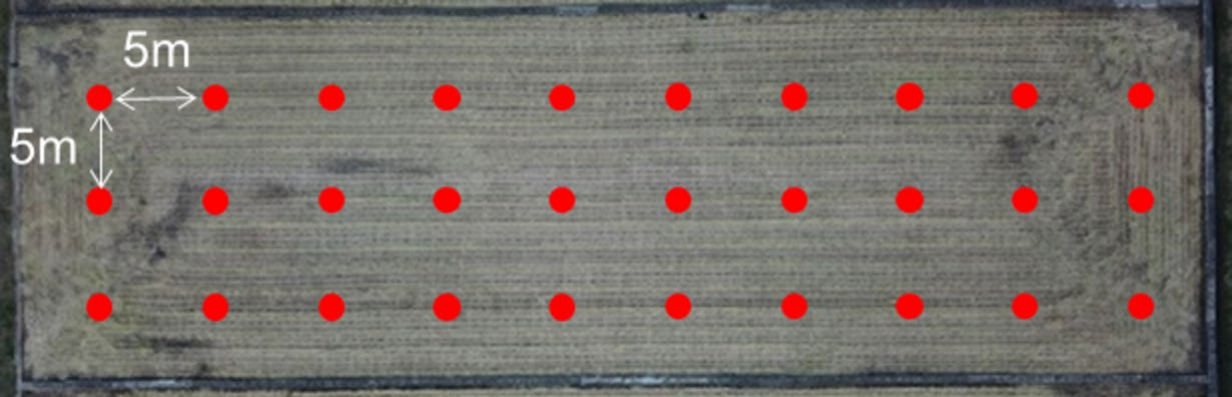
Spots where cone index is measured in test site.

**Fig. 7:**
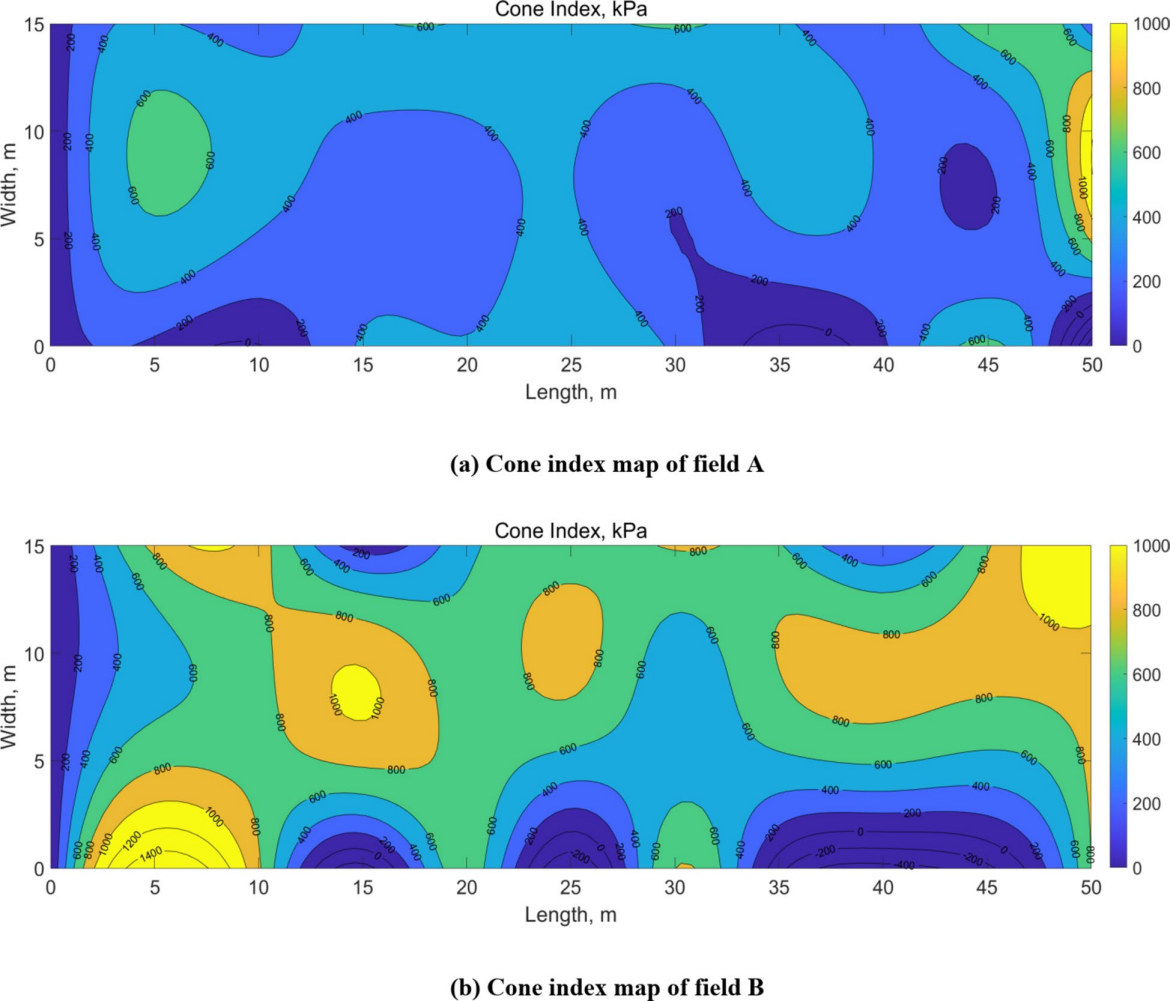
Cone index map generated from average cone index value. (**a**) Cone index map of field A, (**b**) Cone index map of field B.

The cone index at the test site was measured using a bevameter to minimize the influence of the testers’ skill levels^19^. The bevameter is attached to a three-point hitch at the rear of the tractor and is powered by hydraulic pressure from the hydraulic port. The pressure-sinkage device uses hydraulic pressure to lower a cylinder at a constant rate. A cone penetrometer, measuring the cone index, is affixed to the top of a rod connected to the pressure-sinkage tester. Figures 4 and 5 illustrate the bevameter and the cone penetrometer attached to it, respectively. Table 6 specifies the sensors used in the cone penetrometer test. To account for soil property inhomogeneity, the test was conducted on a 5 m grid, as depicted in Fig. 6. The cone index measurement, conducted at a standard penetration rate of 30 mm/s^20^, resulted in a map showing the average cone index from 0 to 15 cm depth at each point. Figure 7(a) shows the cone index map of field A, where the plow tillage was performed, and Fig. 7(b) shows the cone index map of field B, where the rotary tillage was performed.

**Table 6:** Specifications of sensors used in cone penetrometer test.

Item	Model	Specification
Load cell	YG38-500k	Rated capacity: 4905 N Rated output: 2 mV/V ± 0.1% Nonlinearity: 0.05%
LVDT	DWS-12R	Rated range: 1.2 m Nonlinearity: 0.25% Repeatability: 0.008%

#### Test conditions

To accurately measure the maximum traction force for agricultural operations, plow tillage conditions were established at speeds of 2.25 km/h (L3 gear), 3 km/h (L4 gear), 3.4 km/h (M1 gear), and 5 km/h (M2 gear). These speeds are representative of those typically employed for plow tillage operations^21^. The load on the tractor engine increases as the depth of the plow into the soil increases, potentially resulting in an excessive plow load that could stall the engine. To mitigate this, plow load control conditions were implemented through torque and speed control mechanisms:

1. Torque control: This method sets a target plow depth of 150 mm, adjusting the plow’s height to maintain this depth in response to increased soil resistance.
2. Speed control: To prevent engine stalling under heavy plow load, this method allows for an increase in engine torque by reducing the engine speed using the foot throttle. During plow tillage, the foot throttle is fully engaged to maintain full throttle. If excessive load is detected, the operator reduces the engine speed via the foot throttle to decrease the tractor’s travel speed, ensuring consistent plow depth. Plow tillage was conducted in a straight line across field A, starting from one end and stopping at the other.

For measuring maximum PTO torque during agricultural operations, rotary tillage was performed under specific conditions:

1. The engine speed was maintained at a rated speed of 2,200 rpm using the throttle lever.
2. The tractor’s travel speed was set to M1 (3.4 km/h), a common speed for rotary tillage.
3. The PTO was engaged in 3rd gear, ensuring a PTO speed of 1,000 rpm.
4. The target trenching depth was set to 15 cm. Rotary tillage was executed in field B.

To measure the maximum travel speed attainable during agricultural operations, a driving test was conducted with the following parameters:

1. The engine speed was fixed at 2,200 rpm.
2. The tractor’s travel speed was set to H4 (33 km/h). During this test, the tractor accelerated from a standstill to its maximum speed, maintained this speed for a duration, and then decelerated to a stop.

Each operation and gear stage was conducted as a single continuous field run, and the time-series results therefore represent single-run measurements rather than averaged data.

### Workload analysis

The analysis of the tractor’s workload involved calculating the travel speed, traction force (${F}_{t}$), traction power (${P}_{t}$), PTO power (${P}_{PTO}$), and operating points based on the measured data of wheel torque, wheel speed, PTO torque, and PTO speed under various workload conditions. An operating point is defined as the combination of load and speed at which the tractor transmits power, differentiated into traction and PTO operating points depending on the location of output. The traction operating point, determined by the traction force and travel speed at the tractor’s wheels, represents where traction power is transmitted. The PTO operating point, defined by the torque and rotational speed at the PTO shaft, indicates where PTO power is transmitted.

The wheel torque and rotational speed were measured for all four wheels and used to determine the electric tractor’s design requirements. The traction force exerted on the wheels was derived from the wheel torque and radius Eq. (1), and the overall tractor traction force (${F}_{t}$) was calculated as the sum of the tractive forces on all four wheels, as shown in Eq. (2). The tractor speed was computed from the wheel rotational speed using Eq. (3); therefore, the computed speed represents a wheel-based tractor speed and can differ from the actual tractor speed when wheel slip occurs. As the measured wheel speed inherently includes of wheel slip, no additional slip correction was applied in the calculation. Traction power was calculated using Eq. (4) based on the measured wheel torque and rotational speed. Finally, PTO power was calculated as the product of the measured PTO torque and speed, as indicated in Eq. (5).

1 $${F}_{w}=\frac{{T}_{w}}{{r}_{w}}$$

where ${F}_{w}$ is the wheel traction force (N), ${T}_{w}$ is the wheel torque (N‧m), and ${r}_{w}$ is the wheel radius (m).

2 $${F}_{t}={F}_{w,fr}+{F}_{w,fl}+{F}_{w,rr}+{F}_{w,rl}$$

where ${F}_{t}$ is the tractor traction force (N), ${F}_{w,fr}$ is the traction force of the front right wheel (N), ${F}_{w,fl}$ is the traction force of the front left wheel (N), ${F}_{w,rr}$ is the traction force of the rear right wheel (N), and ${F}_{w,rl}$ is the traction force of the rear left wheel (N).

3 $$V=\frac{2\pi{\omega}_{w}{r}_{w}}{60}$$

where $V$ is the wheel-based tractor speed (m/s), and ${\omega}_{w}$ is the wheel speed (rpm).

4 $${P}_{t}=\frac{{T}_{w,fr}{\omega}_{w,fr}+{T}_{w,fl}{\omega}_{w,fl}+{T}_{w,rr}{\omega}_{w,rr}+{T}_{w,rl}{\omega}_{w,rl}}{9548.8}$$

where ${P}_{t}$ is the tractor traction power (kW), ${T}_{w,fr}$ is the wheel torque of the front right wheel (N‧m), ${\omega}_{w,fr}$ is the wheel speed of the front right wheel (rpm), ${T}_{w,fl}$ is the wheel torque of the front left wheel (N‧m), ${\omega}_{w,fl}$ is the wheel speed of the front left wheel (rpm), ${T}_{w,rr}$ is the wheel torque of the rear right wheel (N‧m), ${\omega}_{w,rr}$ is the wheel speed of the rear right wheel (rpm), ${T}_{w,rl}$ is the wheel torque of the rear left wheel (N‧m), and ${\omega}_{w,rl}$ is the wheel speed of the rear left wheel (rpm).

5 $${P}_{PTO}=\frac{{T}_{PTO}{\omega}_{PTO}}{9548.8}$$

where ${P}_{PTO}$ is PTO power (kW), ${T}_{PTO}$ is PTO torque (N‧m), and ${\omega}_{PTO}$ is PTO speed (rpm).

### Determination of design requirements for electric tractors

**Fig. 8:**
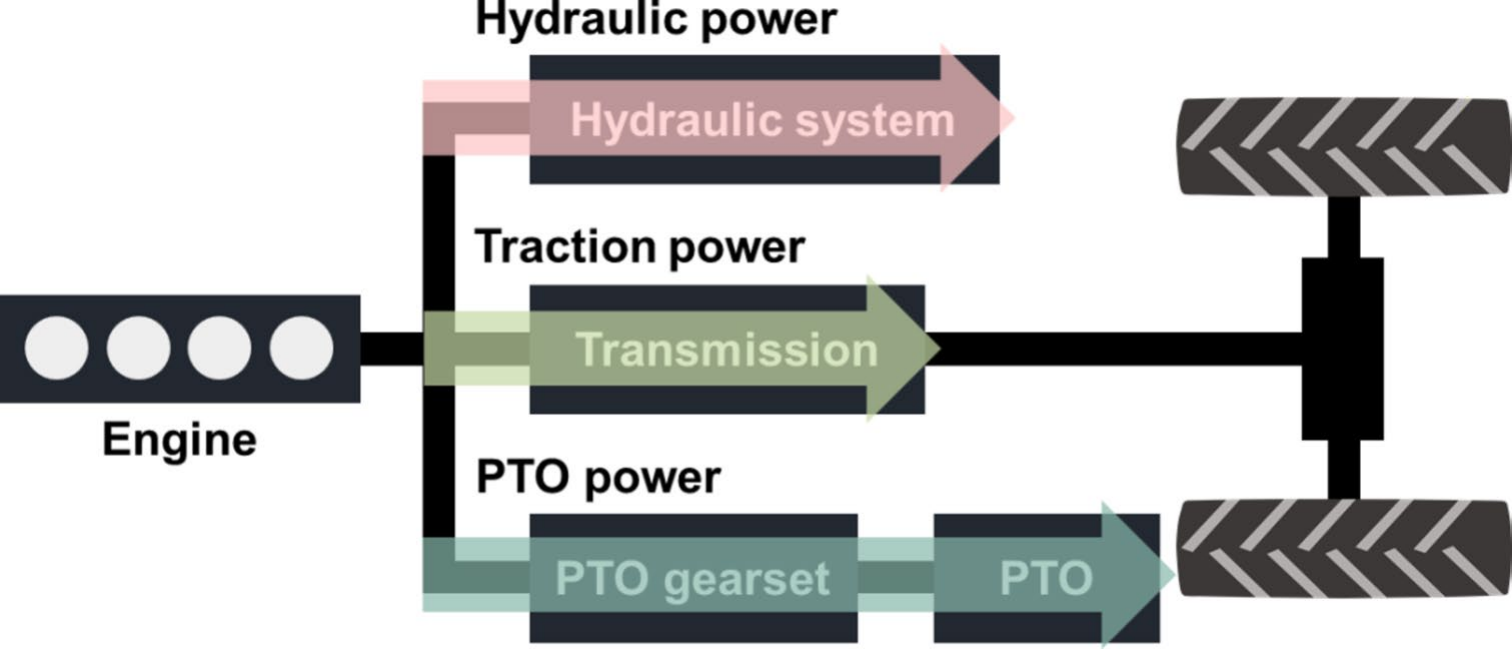
Power transmission paths of tractor.

The power generated by the tractor’s engine is distributed across three main outputs, illustrated in Fig. 8: (1) hydraulic power, which provides energy to hydraulic implements; (2) traction power, which enables the tractor to tow implements; and (3) PTO power, which supplies rotational energy to implements. Given the diverse range of agricultural operations that tractors perform, identifying design requirements necessitates considering not only the peak tractive effort and maximum speed needed for the tasks but also the overall maximum power requirements. Thus, defining the design requirements for an electric tractor involves assessing the maximum traction force, travel speed, and power required by the target operation.

**Fig. 9:**
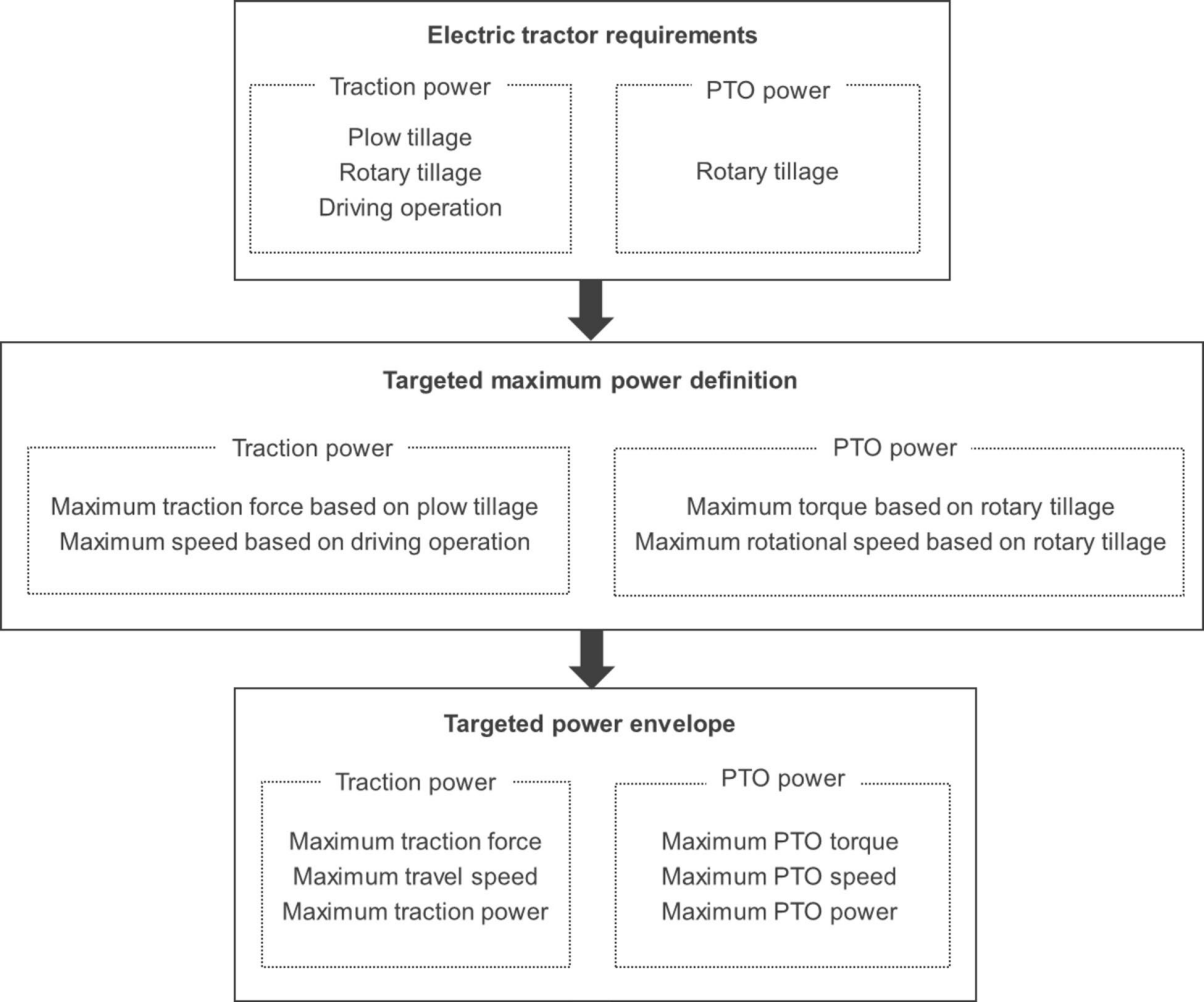
Framework for determining design requirements for electric tractor.

The process for determining the electric-tractor design requirements, as depicted in Fig. 9, is according to the following sequence: Initially, the design requirements are categorized into traction power and PTO power requirements. The traction power requirement is established based on load data from plow tillage, rotary tillage, and driving operations. The PTO power requirement is determined from rotary tillage load data. Hydraulic power is omitted from the electric tractor’s design criteria, assuming the use of a dedicated electric motor for hydraulic functions. Subsequently, in the process of defining the target maximum power, the maximum traction force and travel speed are identified for traction power requirements, while the maximum torque and rotational speed are specified for PTO power requirements. These metrics are derived from plow tillage (maximum traction force), driving operations (maximum travel speed), and rotary tillage (maximum torque and rotational speed). The final step involves the targeted power envelope process, where the power envelope curves for both traction and PTO power requirements are established. The traction power’s envelope curve is defined by a curve that meets the tractor’s maximum traction force, travel speed, and traction power. Similarly, the PTO power’s envelope curve is determined by a curve that accommodates the maximum torque of the PTO shaft, maximum rotational speed, and maximum PTO power^22^.

## Results

### Workload analysis

#### Plow tillage operation

**Fig. 10:**
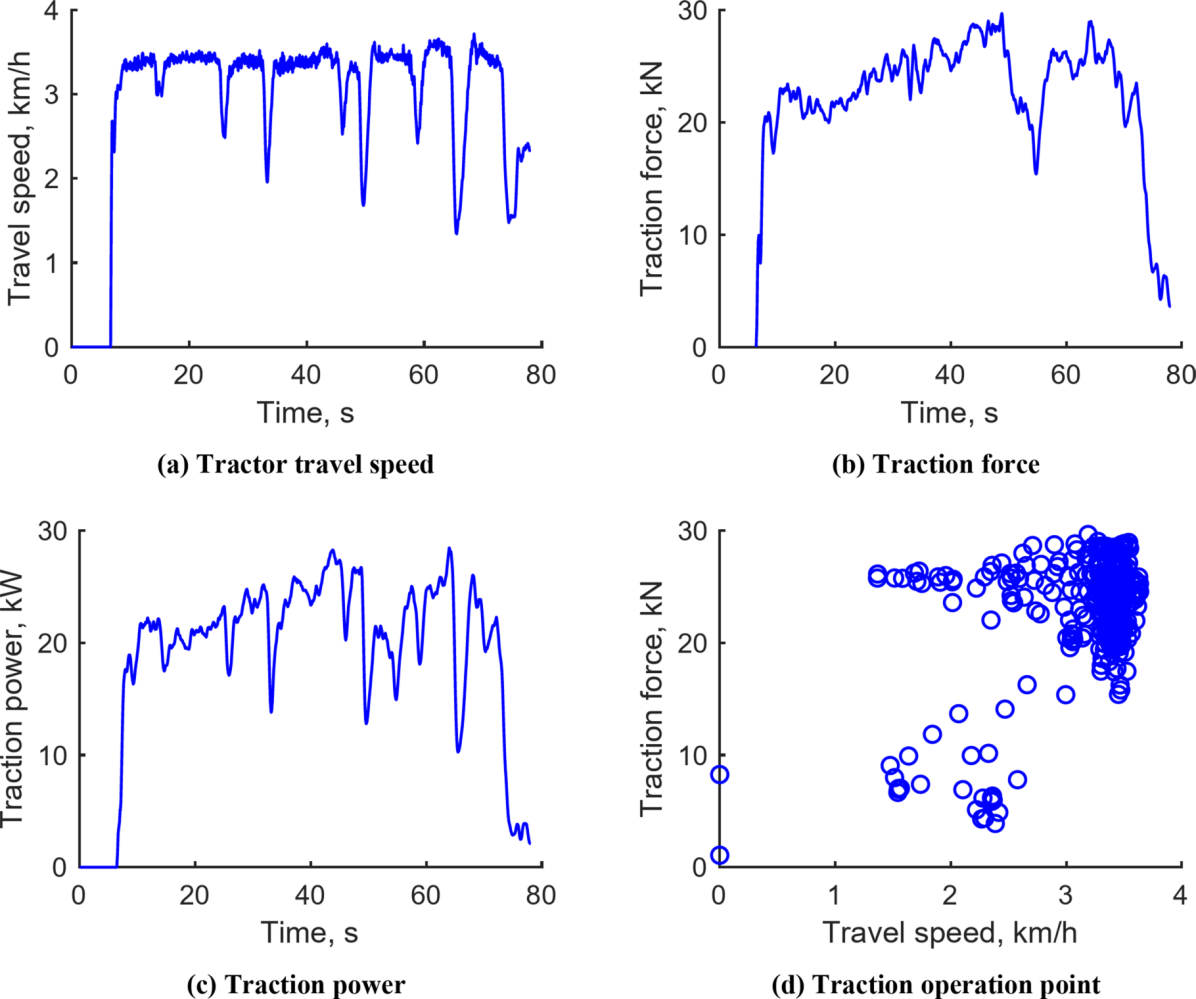
Representative workload data during plow tillage operation under M1 gear. (**a**) Tractor travel speed, (**b**) Traction force, (**c**) Traction power, (**d**) Traction operation point.

Figure 10 illustrates the plow tillage operation results conducted in M1 mode, aiming for a target depth of 150 mm under varying engine speeds. The tractor’s travel speed ranged between 1.34 and 3.72 km/h, averaging 3.24 km/h. The speed was significantly reduced in certain sections due to factors such as plow load and slip. The traction force exhibited a range from 15.40 to 29.71 kN, with an average of 22.65 kN. Traction power varied between 10.24 and 28.44 kW, with an average of 22.04 kW, calculated by the product of tractor travel speed and traction force, indicating lower values in sections where the tractor’s speed decreased. The traction operating point revealed that tractor travel speeds were predominantly between 1 and 3.5 km/h, with traction forces mainly distributed between 18 and 30 kN. Detailed plow tillage test data for the L3, L4, and M2 stages are provided in Supplementary Figs. S1–S3.

#### Rotary tillage operation

**Fig. 11:**
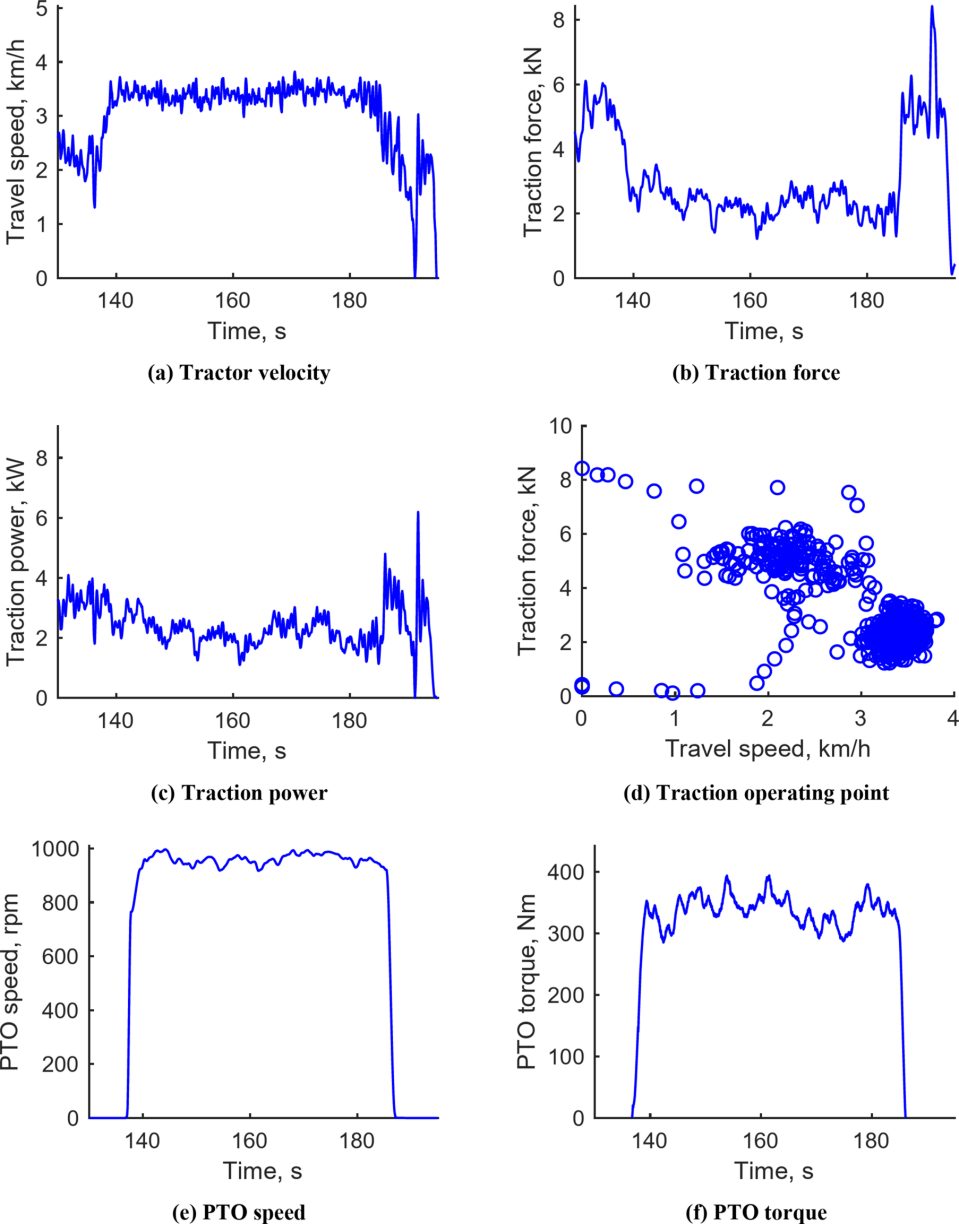

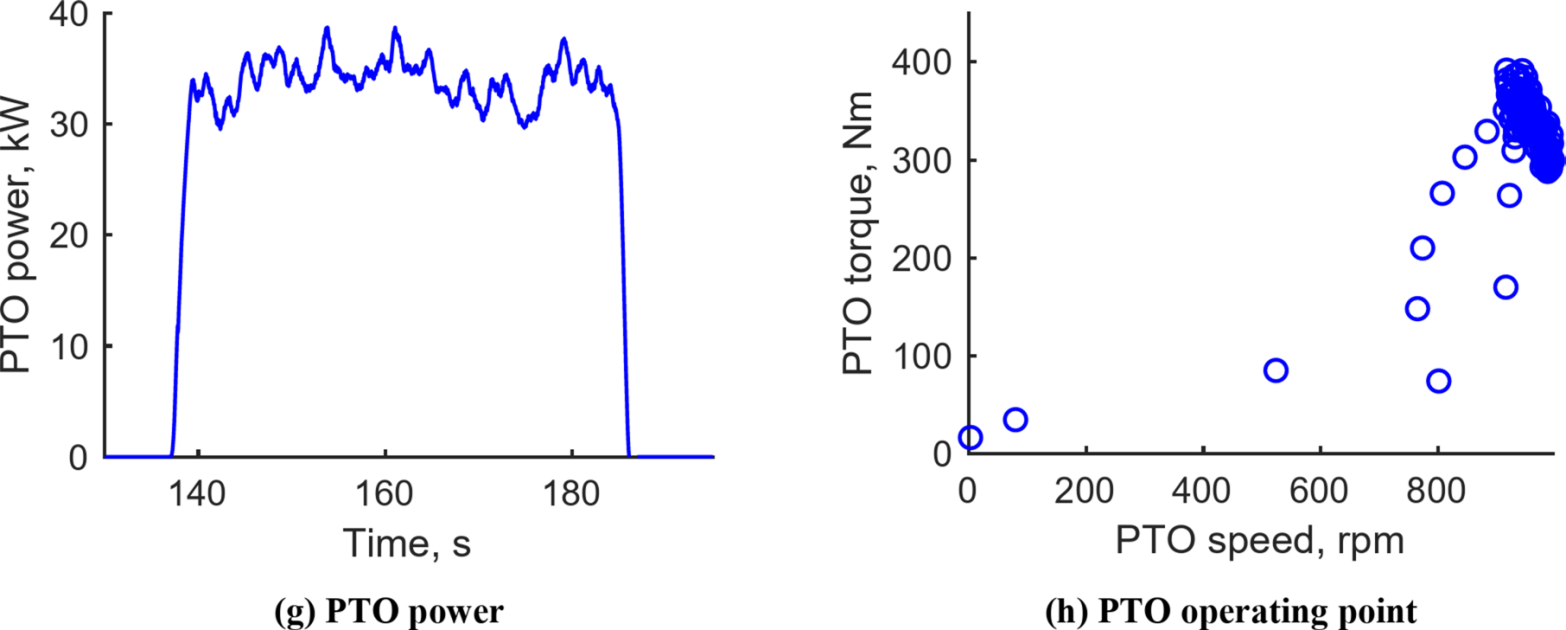
Representative workload data during rotary tillage operation under M1 & PTO 3rd gears. (**a**) Tractor velocity, (**b**) Traction force, (**c**) Traction power, (**d**) Traction operating point, (**e**) PTO speed, (**f**) PTO torque, (**g**) PTO power, (**h**) PTO operating point.

Figure 11 presents the results from the rotary tillage conducted with M1 gear, PTO 3, an engine speed of 2200 rpm, and a target depth of 150 mm. The tractor’s travel speed ranged from 2.99 to 3.82 km/h, with an average of 3.39 km/h. Traction forces varied from 1.23 to 8.43 kN, with an average of 3.06 kN. During the operation, the traction force primarily ranged between 1.28 and 3.30 kN, correlating with tractor speeds of 3 to 3.82 km/h. PTO speed ranged from 807.51 to 1008.73 rpm, marginally decreasing as PTO load increased. PTO torque varied between 200.47 and 441.86 N‧m, averaging 296.97 N‧m, while PTO power spanned from 20.69 to 39.63 kW, with an average of 24.02 kW. At the PTO operating point, speeds mostly ranged from 800 to 1000 rpm and torque from 200 to 400 N‧m. Notably, the average traction power during rotary tillage was 2.57 kW, and the average PTO power was 24.02 kW; thus, traction power constituted 9.67% of the total power requirement. This highlights the importance of considering both PTO and traction power in determining electric-tractor design requirements. Data for rotary tillage with M1 gear, an average travel speed of 3.4 km/h, and 3 PTOs are detailed in Supplementary Fig. S4.

#### Driving operation

**Fig. 12:**
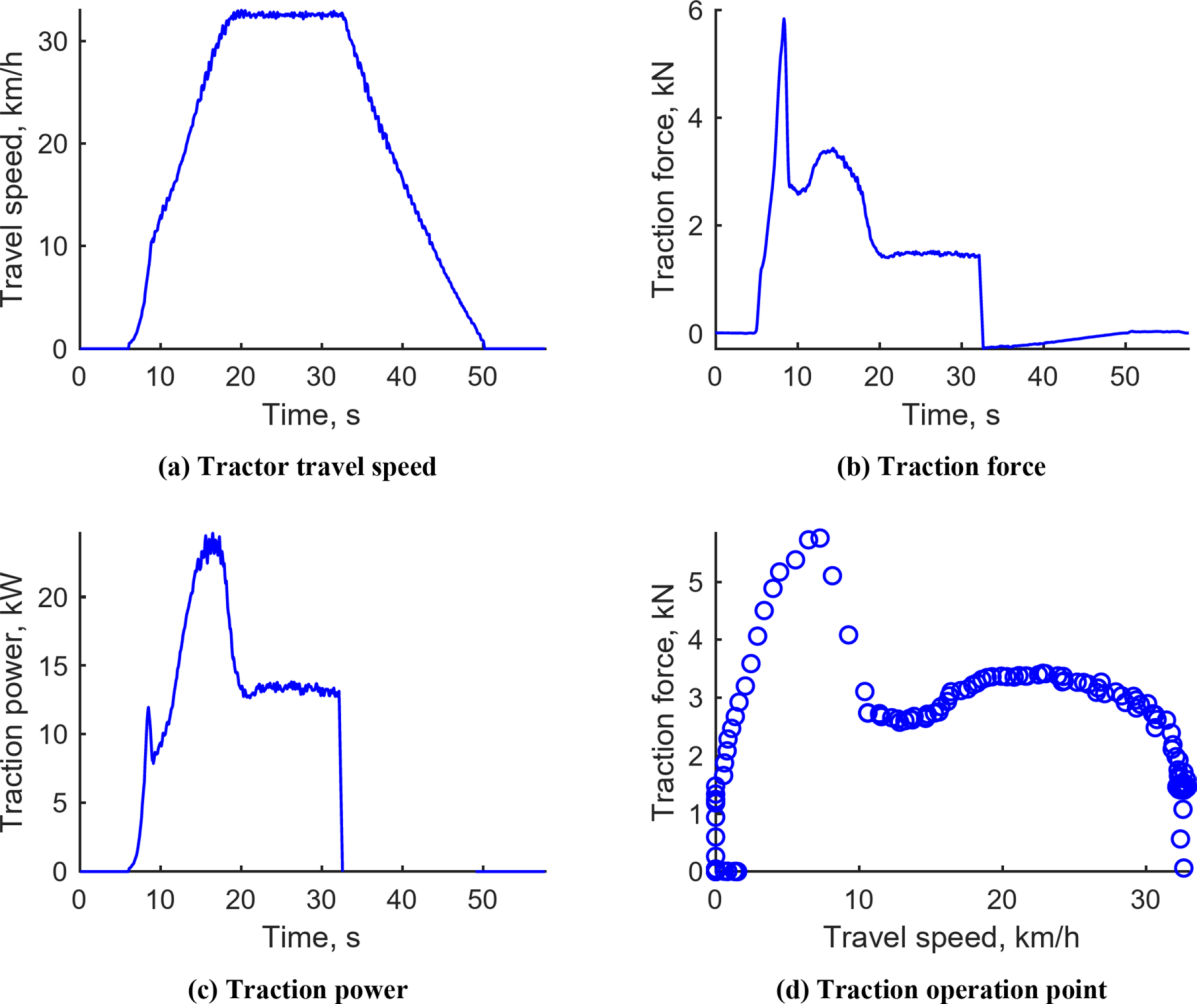
Representative workload data during driving operation in H4 gear. (**a**) Tractor travel speed, (**b**) Traction force, (**c**) Traction power, (**d**) Traction operation point.

Figure 12 presents the results from the driving operation conducted in H4 gear at an engine speed of 2200 rpm. The tractor was tasked with reaching a target travel speed of 33 km/h. It achieved a maximum travel speed of 33.00 km/h, with a maximum traction force of 5.84 kN observed during the acceleration phase. The peak traction power recorded during this phase was 24.65 kW, necessary to attain the maximum speed. The traction force was relatively low compared to other agricultural operations, attributed to the tractor operating at high speeds without bearing any additional agricultural load.

#### Tractor operating point

**Fig. 13:**
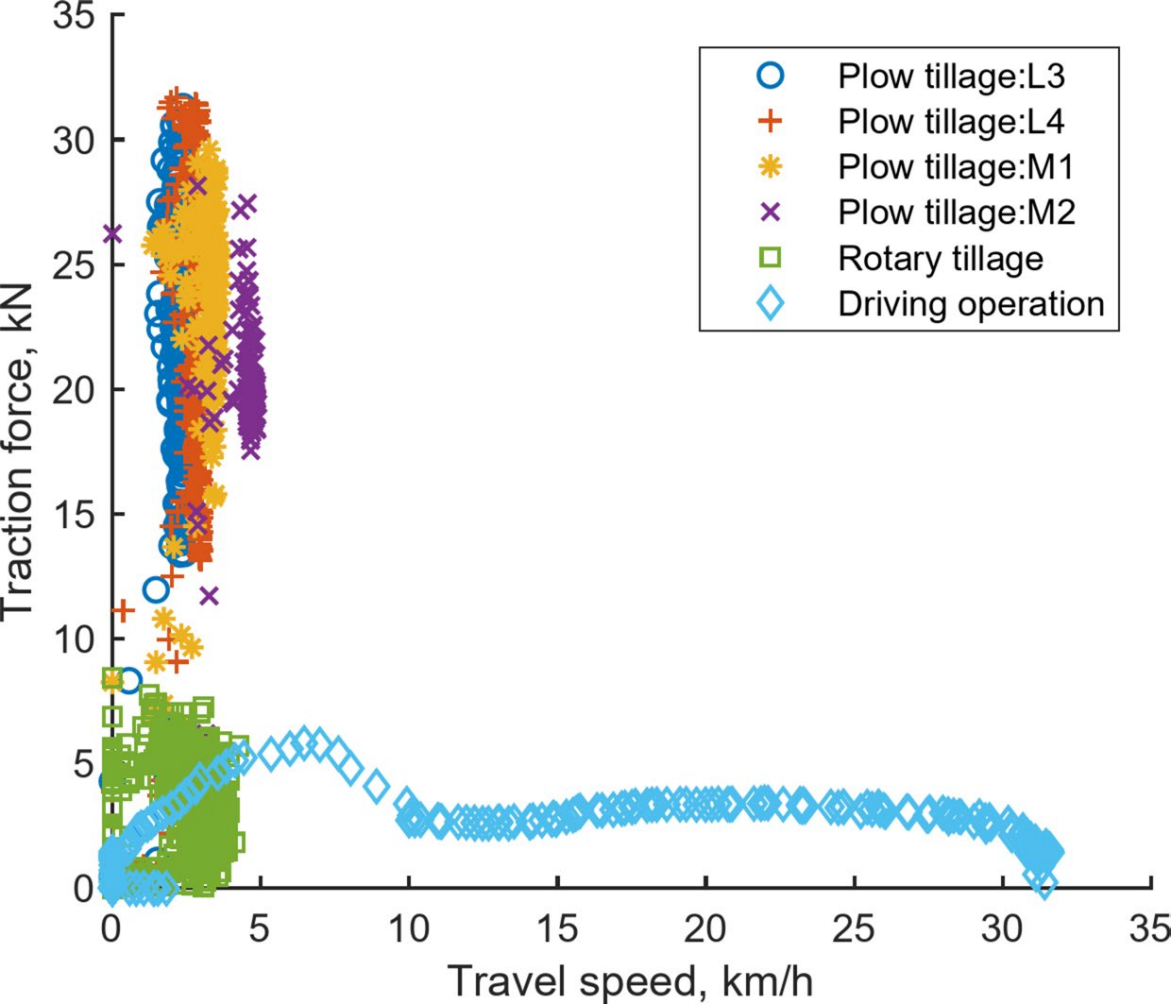
Traction operating point based on field test data.

**Fig. 14:**
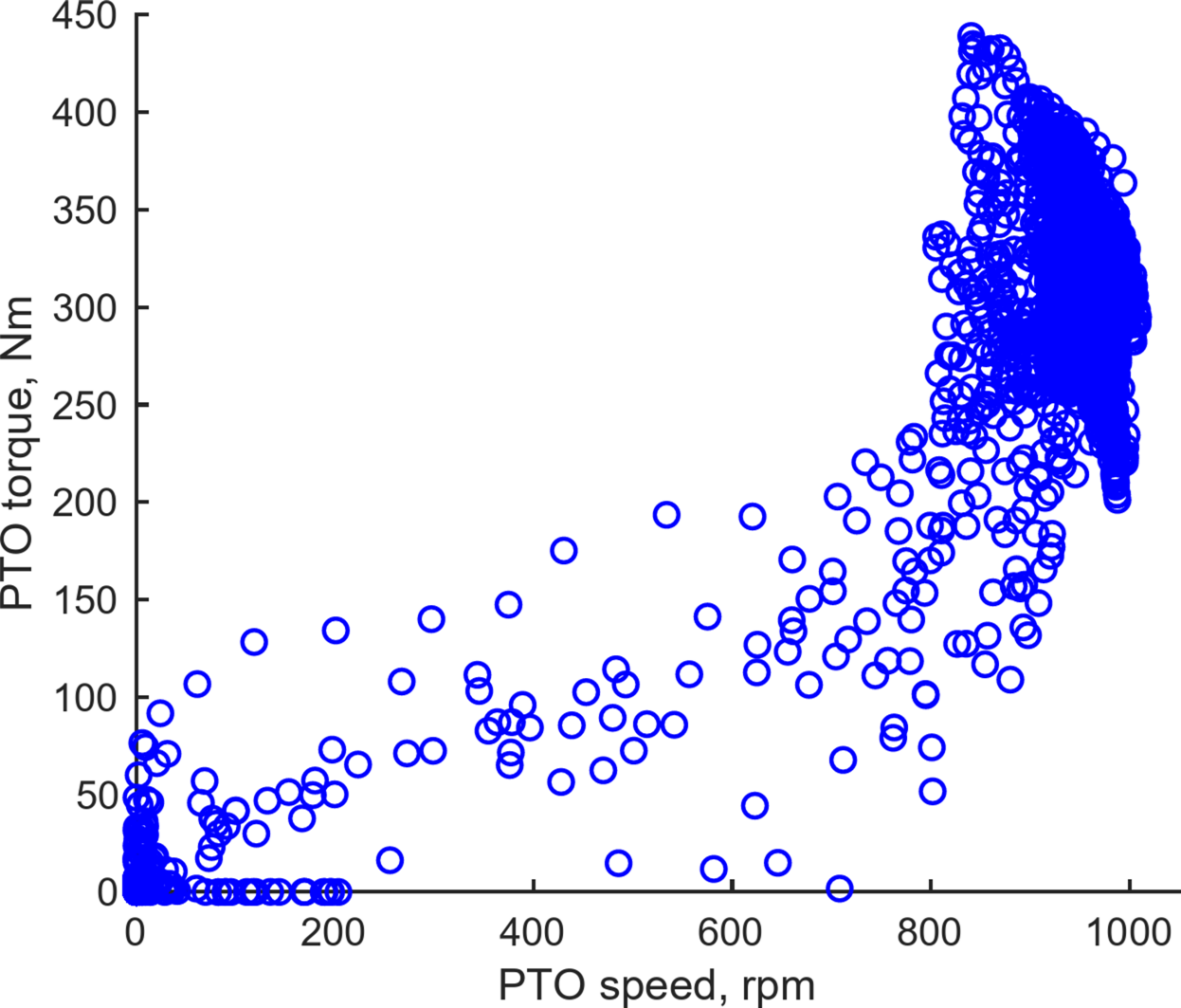
PTO operating point based on field test data.

Figure 13 delineates the traction operating points for various operations, including plow tillage (L3, L4, M1, M2 gears), rotary tillage, and driving, derived from agricultural workload test data. The highest tractive force recorded during plow tillage was 31.70 kN, whereas the maximum travel speed during the driving operation reached 33.00 km/h. Figure 14 illustrates the PTO operating point for rotary tillage based on the collected data, revealing a maximum PTO torque of 441.86 N‧m and a peak PTO speed of 1008.73 rpm.

### Determination of design requirements for electric tractors

The development of the power envelope curve for traction power was based on analyzing agricultural workload data, including traction force, travel speed, and traction power, under conditions of maximum traction force, travel speed, and traction power. Table 7 summarizes the traction power requirements for various agricultural operations. For plow tillage, the maximum traction force reached 31.70 kN in L4 gear, the highest travel speed was 4.60 km/h in M2 gear, and the peak traction power was 35.01 kW, also in M2 gear. During the driving operation, the peak traction force was 5.84 kN, the maximum travel speed was 33.00 km/h, and the maximum traction power was 24.64 kW. For rotary tillage, the traction force peaked at 8.43 kN, with a maximum travel speed of 5.03 km/h and a maximum traction power of 9.05 kW. Thus, the greatest traction force was derived from plow tillage, the highest travel speed from the driving operation, and the maximum traction power from plow tillage. Based on this data, the design requirements for traction power can be established to define the power envelope curve for traction power as follows:

1. Condition for maximum traction force: The highest traction force recorded was 31.70 kN during plow tillage in L4 gear. To facilitate effective plow tillage, the electric tractor’s design must account for the maximum traction force across the travel speed range where plowing is viable. The optimal travel speed for applying this maximum traction force is 4.60 km/h. Thus, the target maximum traction force and travel speed are set at 31.70 kN and 4.60 km/h, respectively, leading to a targeted power of 40.50 kW.

2) Condition for maximum travel speed: Achieving the tractor’s maximum speed requires setting a target maximum speed of 33.00 km/h, with a traction force of 1.47 kN and a traction power of 13.45 kW.

3) Condition for maximum traction power: The tractor should support plow tillage under conditions that generate the maximum power. Here, the target traction force is 27.95 kN, and the target travel speed is 4.51 km/h, resulting in a target traction power of 35.01 kW.

4) Condition for PTO operation driving: The tractor must also perform efficiently at target travel speeds during PTO operations such as rotary tillage. For this, the target traction force is set at 8.43 kN, with a travel speed of 5.03 km/h and a maximum traction power of 9.05 kW.

**Fig. 15:**
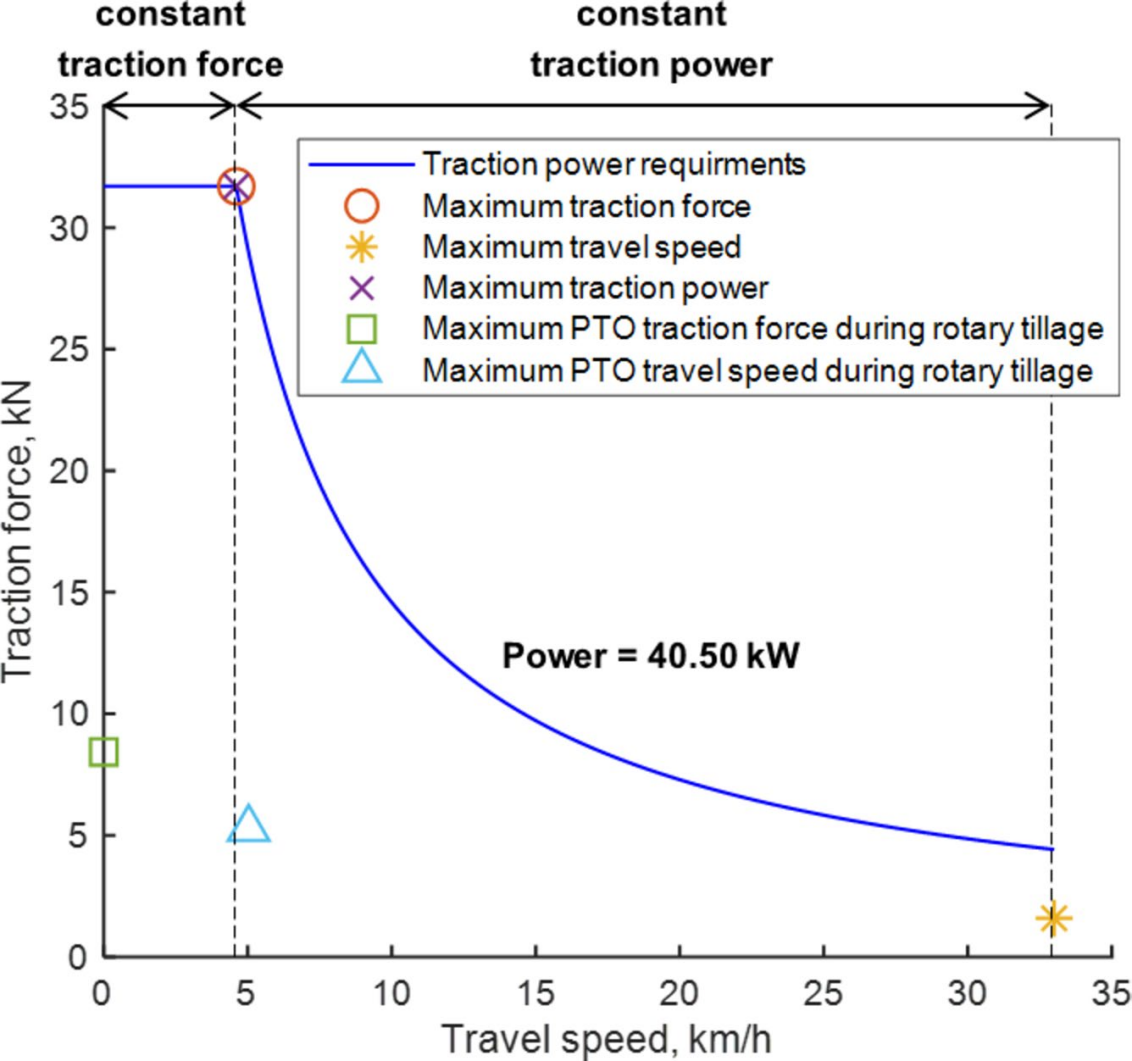
Traction power envelope determined from design requirements.

In setting the design requirements for traction power, the target maximum traction force was established at 31.70 kN, achievable at a travel speed of 4.6 km/h. The target maximum travel speed was set at 33.00 km/h, with the target maximum traction power set at 40.50 kW. Table 8 outlines the traction power design requirements for electric tractors, which informed the development of the traction power envelope curve. This curve is segmented into two distinct regions: a constant traction force region, where the maximum traction force is consistently applied, and a constant traction power region, characterized by the application of steady traction power. Within the constant traction force region, the maximum traction force of 31.70 kN is sustained up to the maximum travel speed of 4.60 km/h. In the constant traction power region, the traction force and travel speed are adjusted to maintain the maximum power of 40.50 kW stipulated by the traction power design requirements, extending up to the maximum travel speed of 33.00 km/h. Figure 15 depicts the traction power envelope curve derived from these design requirements. The overlay of the measured traction operating points and the proposed traction requirement envelope is presented in Supplementary Fig. S5.

**Table 7:** Traction power requirements for agricultural operation.

Work	Condition	Traction power		
		Force (kN)	Speed (km/h)	Power (kW)
Plow	Maximum traction force	**31.70**	2.81	24.74
	Maximum travel speed	4.55	**4.60**	5.81
	Maximum traction power	27.95	4.51	**35.01**
Driving	Maximum traction force	**5.84**	6.90	11.20
	Maximum travel speed	1.47	**33.00**	13.45
	Maximum traction power	3.06	29.03	**24.64**
Rotary	Maximum traction force	**8.43**	0.02	0.05
	Maximum travel speed	5.27	**5.03**	7.36
	Maximum traction power	7.59	4.29	**9.05**

**Table 8:** Traction power design requirements for electric tractor.

Condition	Traction power		
	Force (kN)	Speed (km/h)	Power (kW)
Maximum traction force	**31.70**	4.60	40.50
Maximum travel speed	1.47	**33.00**	13.45
Maximum traction power	31.70	4.60	**40.50**

The power envelope curve for PTO power was established based on agricultural workload data, including PTO torque, PTO speed, and PTO power, under conditions of maximum PTO torque, speed, and power. Table 9 summarizes the PTO power requirements for agricultural operations. Specifically, in rotary tillage, the maximum torque reached 441.86 N‧m, the peak rotational speed was 1008.73 rpm, and the highest power was 39.63 kW. From this data, the PTO power design requirements to formulate the PTO power envelope curve were identified as follows:

1. Condition for maximum PTO power: The tractor should support rotary tillage under workload conditions that yield the highest PTO power. Accordingly, the target PTO speed, torque, and power were set at 867.55 rpm, 436.22 N‧m, and 39.63 kW, respectively.
2. Condition for PTO operating point region: Rotary operations typically utilize PTO speeds of 540, 750, and 1000 rpm, corresponding to a three-stage PTO speed setting^21^. To enable effective rotary work across these PTO-speed settings, the maximum required PTO power was defined as 39.63 kW based on the measured PTO workload data obtained under the 3rd-speed setting (1000 rpm). The corresponding torque targets were then specified for each PTO-speed setting: 700.77 N·m for the 1st-speed setting (540 rpm), 504.55 N·m for the 2nd-speed setting (750 rpm), and 378.41 N·m for the 3rd-speed setting (1000 rpm). The torque targets for the 1st- and 2nd-speed settings were obtained as constant-power-equivalent torques derived from the same maximum PTO power requirement, rather than directly measured torques at 540 rpm and 750 rpm.

**Fig. 16:**
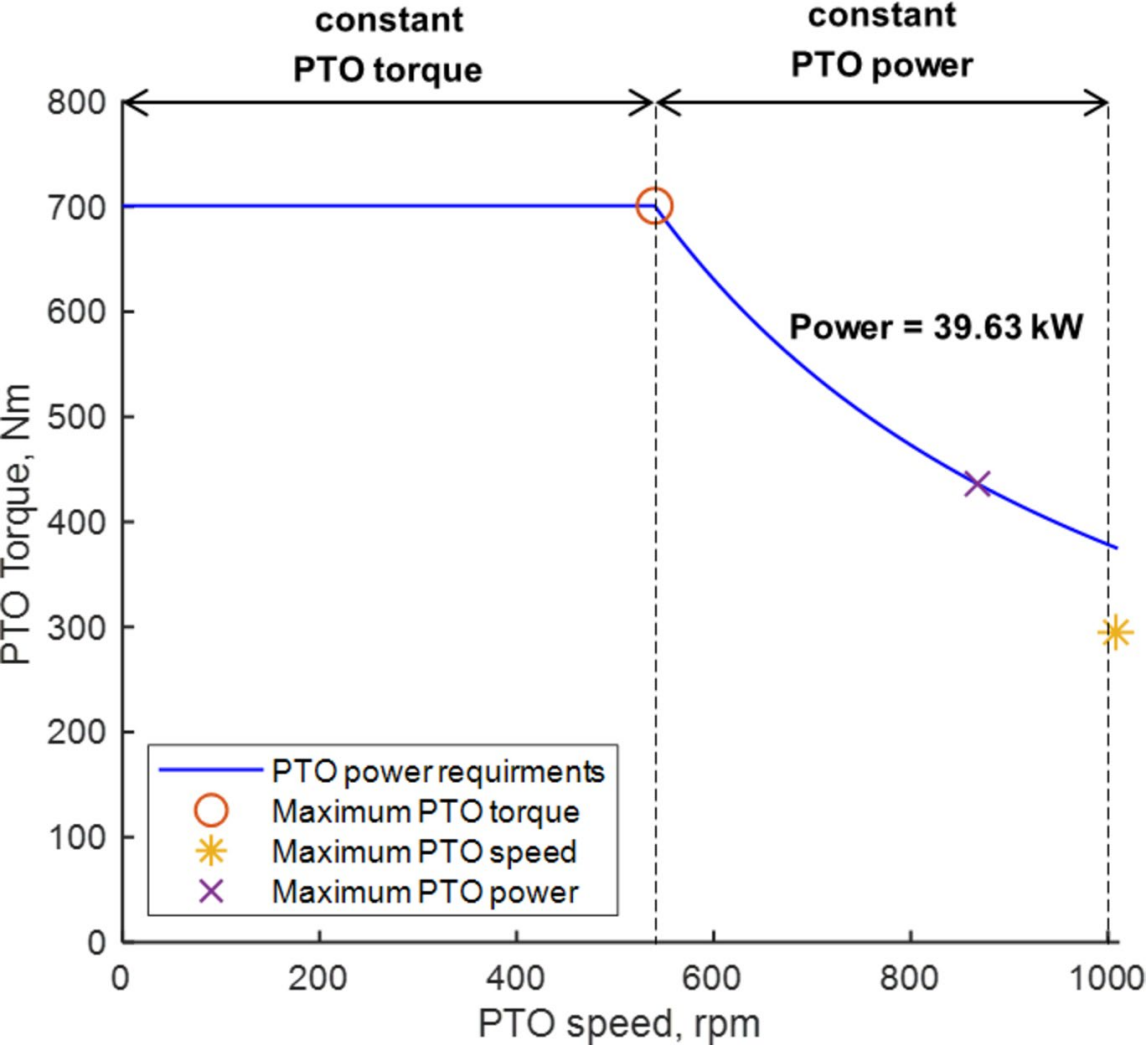
PTO power envelope determined from design requirements.

The PTO power design requirements were determined as a maximum PTO torque of 700.77 N‧m, a maximum PTO speed of 1000 rpm, and a maximum PTO power of 39.63 kW. Table 10 presents the PTO power design requirements for the electric tractor, which informed the development of the PTO power envelope curve. This curve is segmented into a constant PTO torque region, maintaining the highest torque of 700.77 N‧m up to a PTO speed of 540 rpm, and a constant PTO power region, where PTO torque and speed are adjusted to sustain a constant maximum power of 39.63 kW up to the peak PTO speed of 1008.73 rpm. Figure 16 illustrates the PTO power envelope curve based on these design requirements. The overlay of the measured PTO operating points and the proposed PTO requirement envelope is presented in Supplementary Fig. S6.

**Table 9:** PTO power requirements for agricultural operation.

Work	Condition	PTO power		
		Torque (N‧m)	Rotational speed (rpm)	Power (kW)
Rotary	Maximum PTO torque	**441.86**	840.95	38.91
	Maximum PTO speed	293.84	**1008.73**	31.04
	Maximum PTO power	436.22	867.55	**39.63**

**Table 10:** PTO power design requirements for electric tractor.

Condition	PTO power		
	Torque (*N*‧m)	Rotational speed (rpm)	Power (kW)
Maximum PTO torque	**700.77**	540	39.63
Maximum PTO speed	293.84	**1008.73**	31.04
Maximum PTO power	436.22	867.55	**39.63**

## Discussion

### Comparison between different powertrain configurations of electric tractors

Based on the design requirements derived from agricultural workload data, it was concluded that the design of electric tractors must account for both traction power, necessary for plow tillage and driving operations, and PTO power, crucial for PTO operations such as rotary tillage. Consequently, three powertrain configurations were proposed to manage two power outputs (traction and PTO) within the electric-tractor powertrain^6,13,23,24^: (1) single-motor type, (2) dual-motor & power-separated type, and (3) dual-motor & power-assist type.

All three configurations incorporate a dedicated electric motor for the hydraulic system. They differ in the generation of traction and PTO power—either by a single motor (single-motor type) or by two motors (dual-motor and power-separated type, dual-motor and power-assist type).

In this study, the traction power design requirement and PTO power design requirement were set to 40.50 kW and 39.63 kW, respectively. During rotary tillage, the tractor requires 39.63 kW of PTO power with an additional traction power demand of 9.05 kW for travel. Therefore, depending on the powertrain configuration and operating mode, the required power demand at the traction wheels and PTO shaft can differ among configurations; for operating modes requiring both outputs simultaneously, it is defined as the sum of the traction and PTO power demands. The required motor power is calculated by accounting for the overall mechanical powertrain efficiency and motor efficiency as follows:

6 $${P}_{req,motor}=\frac{{P}_{req,out}}{{\eta}_{tm}\cdot{\eta}_{motor}}$$

Where, ${P}_{req,motor}$ is the required motor power of powertrain configuration, ${P}_{req,out}$ is the required power demand at the traction wheels and PTO shaft of powertrain configuration and operating mode, ${\eta}_{tm}$ is the mechanical powertrain efficiency of powertrain configuration and ${\eta}_{motor}$ is the motor efficiency. The overall mechanical powertrain efficiency, ${\eta}_{tm}$​, is powertrain configuration and operating-point-dependent because it reflects the specific power path and the number and type of drivetrain elements involved. Accordingly, once a detailed drivetrain configuration is specified, the efficiency of the transmission​ can be estimated as the product of component efficiencies along the corresponding power path.

**Fig. 17:**
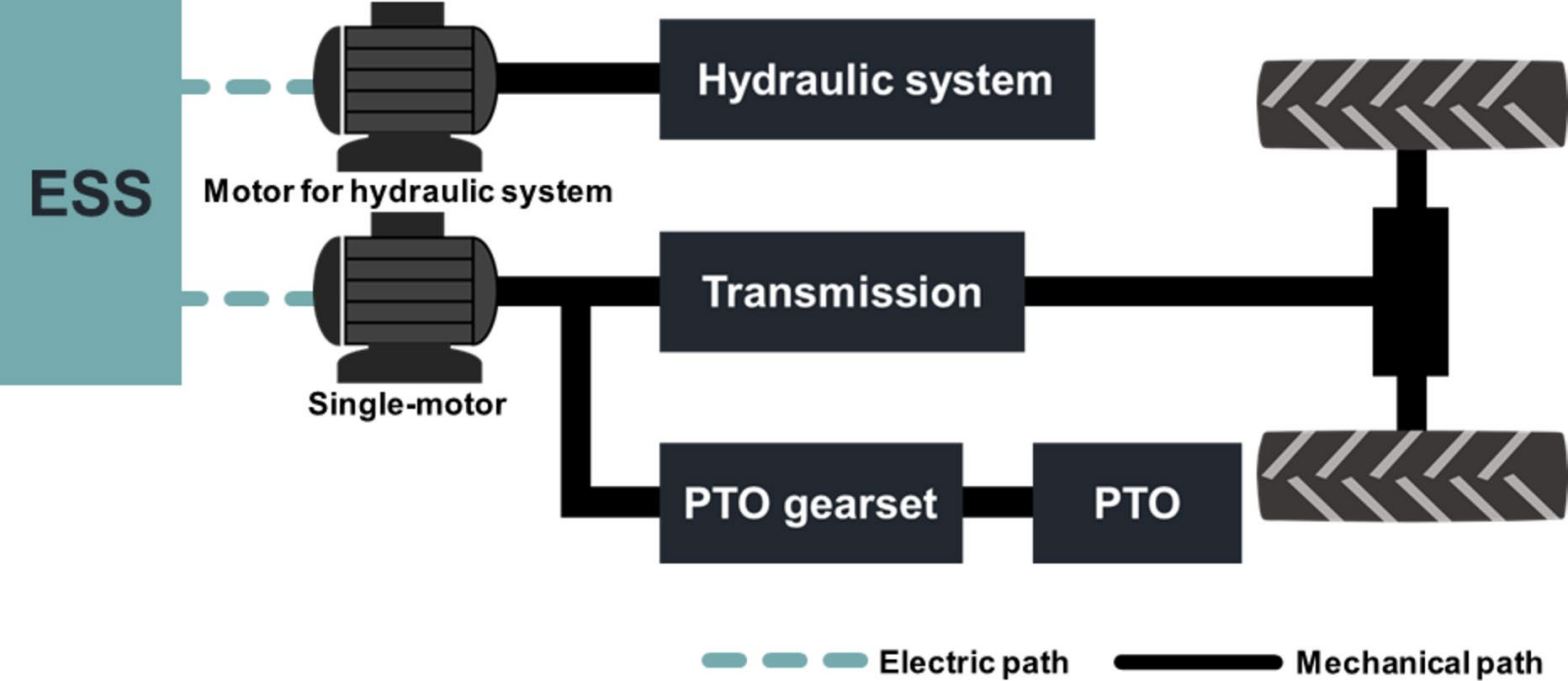
Single-motor type electric-tractor powertrain configuration.

The single-motor type configuration is similar to the structure of traditional combustion engine tractors, generating both traction and PTO power—excluding hydraulic power—using a single electric motor, as illustrated in Fig. 17. This motor must fulfill the traction power design requirement of 40.50 kW and accommodate 39.63 kW for PTO power and an additional 9.05 kW for traction power required during rotary tillage. Therefore, the required power demand at the traction wheels and PTO shaft of single-motor type configuration is 48.68 kW during rotary tillage, and the required motor power is calculated using Eq. (6), accounting for the mechanical powertrain efficiency and motor efficiency. However, this configuration necessitates a complex multispeed transmission to deliver the power needed for various agricultural operations, meet the maximum traction force requirement of 31.70 kN, achieve a maximum travel speed of 33.00 km/h, and support rotary tillage at varying speeds. As a result, the single-motor type configuration leads to a more complex tractor powertrain structure and reduces power transmission efficiency. Nonetheless, control of the powertrain is considered simpler due to the single-motor system^25^.

**Fig. 18:**
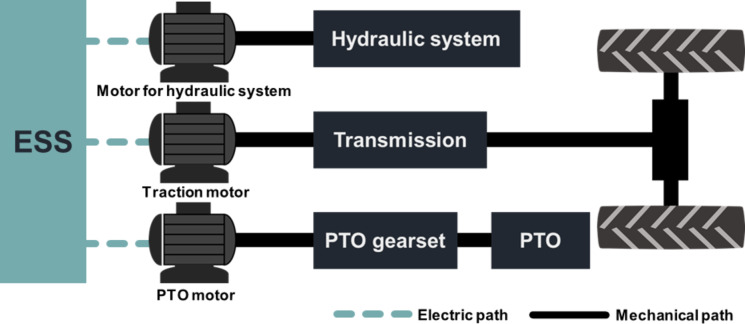
Dual-motor & power-separated type electric-tractor powertrain configuration.

The dual-motor & power-separated type configuration for electric tractors employs two distinct motors: one for traction power and another for PTO power. This setup effectively segregates the traction and PTO functions, with each motor dedicated to a specific task, as illustrated in Fig. 18. The required power demand at the traction wheel is 40.50 kW and the required power demand at the PTO shaft is 39.63 kW. The corresponding required motor input power for the traction motor and PTO motor is calculated using Eq. (6). This configuration allows for a simpler transmission design with fewer gears than that required for the single-motor configuration, facilitating the achievement of a maximum traction force of 31.70 kN for plow operations and a maximum travel speed of 33.00 km/h for driving operations. Furthermore, it enables the independent operation of tractor travel speed and PTO torque by simultaneously activating the PTO and traction motors during rotary tillage. As a result, this powertrain configuration simplifies the tractor’s overall structure and enhances power transmission efficiency. Additionally, the control complexity is reduced due to the independent management of the two motors.

**Fig. 19:**
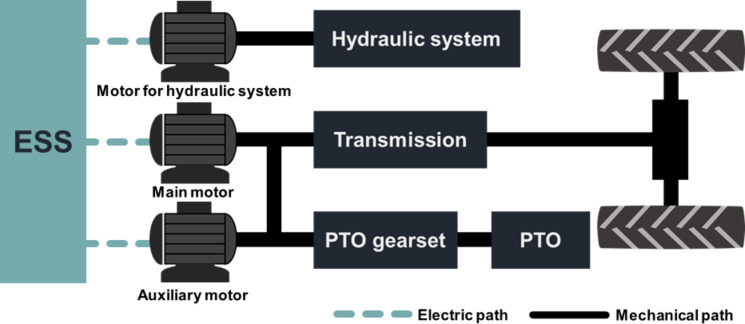
Dual-motor & power-assist type electric-tractor powertrain configuration.

The dual-motor & power-assist type configuration for electric tractors employs a system where two motors—a main motor and an auxiliary motor—are utilized to generate the necessary traction and PTO power for agricultural operations, as depicted in Fig. 19. According to the design requirements, 40.50 kW of traction power is needed for plow tillage, and for rotary tillage, 39.63 kW of PTO power along with 9.05 kW of traction power is required. In this setup, the main motor acts as the primary power source while the auxiliary motor serves as a secondary power source, leading to three distinct driving modes: (1) exclusive use of traction power, (2) exclusive use of PTO power, and (3) concurrent use of both traction and PTO power. In traction-only mode, the combined power of both motors is directed to the drive wheels. In PTO-only mode, the main motor alone provides power. During simultaneous use, the main motor supplies PTO power, while the auxiliary motor provides traction power. Consequently, the motor capacity requirements are a minimum of 39.63 kW for the main motor and 9.05 kW for the auxiliary motor. Therefore, the required motor power for the main motor and auxiliary motor is calculated using Eq. (6). This dual-motor configuration facilitates a simplified transmission design, similar to the dual-motor & power-separated type, offering a simpler solution than the single-motor configuration to meet the maximum traction force of 31.70 kN and a maximum travel speed of 33.00 km/h. Moreover, it enables the simultaneous operation of the PTO and traction motors during rotary tillage, allowing for the generation of desired PTO torque at the required tractor travel speed. Although the transmission structure in the dual-motor & power-assist type is relatively simple, it becomes more complex than the power-separated type due to the inclusion of mechanisms such as clutches to engage and disengage the power from the main and auxiliary motors. While the transmission efficiency is expected to be high owing to the simplified structure, the system demands sophisticated control to integrate, distribute, and disconnect power based on the power demands of various agricultural operations.

Table 11 provides a comparison of the structural complexity, powertrain efficiency, and control difficulty associated with three distinct powertrain configurations for electric tractors. Each configuration presents a unique set of advantages and challenges:

- Single-motor type: This configuration benefits from a lower control difficulty and the ability to utilize the powertrain structure of a traditional ICE tractor. However, it has the disadvantages of a complex transmission structure and reduced power transmission efficiency.
- Dual-motor & power-separated type: This setup independently generates traction and PTO power, offering a simpler transmission structure and moderate control difficulty. Its drawback lies in the aggregate power of the traction and PTO motors, which significantly exceeds the design requirements, leading to an overall larger motor power requirement compared to other configurations.
- Dual-motor & power-assist type: This configuration combines two motors to produce both traction and PTO power, allowing for the selection of motors that meet the minimum power requirements as specified by the design. The challenge with this configuration is the need for operational modes to integrate and segregate power, which complicates the powertrain structure and increases control difficulty.

The proposed power envelopes can be used in follow-up studies to relate workload-based design requirements to battery sizing (energy capacity and discharge capability) and motor thermal constraints.

**Table 11:** Performance comparison of three powertrain configurations suggested.

Powertrain configuration	Required motor power (kW)		Structural complexity	Powertrain efficiency	Control difficulty
Single-motor type	Motor	$\frac{48.68}{{\eta}_{tm}\cdot{\eta}_{motor}}$	High	Low	Low
Dual-motor & power-separated type	Traction motor	$\frac{40.50}{{\eta}_{tm}\cdot{\eta}_{motor}}$	Low	High	Medium
	PTO motor	$\frac{39.63}{{\eta}_{tm}\cdot{\eta}_{motor}}$			
Dual-motor & power-assist type	Main motor	$\frac{39.63}{{\eta}_{tm}\cdot{\eta}_{motor}}$	Medium	High	High
	Auxiliary motor	$\frac{9.05}{{\eta}_{tm}\cdot{\eta}_{motor}}$			

## Conclusions

This study established the design requirements for an electric tractor by assessing the workloads generated during various agricultural operations with a target-specification ICE tractor. The research analyzed the characteristics of proposed electric-tractor powertrain configurations through agricultural workload tests conducted in plow tillage, rotary tillage, and driving operations. The data was separately evaluated for traction power and PTO power, leading to the specification of design requirements for each. These requirements were conceptualized as a power envelope curve, identifying the speed–force characteristics essential for electric-tractor design. For traction power, the maximum traction force was identified at 31.70 kN, the maximum travel speed at 33.00 km/h, and the maximum traction power at 40.50 kW, specifically under L4 plow tillage conditions. For PTO power, the peak torque was set at 700.77 N‧m for the 1st-speed of PTO, with a maximum rotational speed of 1008.73 rpm and a maximum PTO power of 39.63 kW.

Upon defining the electric-tractor design requirements, three powertrain configurations were proposed: (1) single-motor type, (2) dual-motor & power-separated type, and (3) dual-motor & power-assist type. The study delved into comparing these configurations, focusing on motor requirements, structural complexity, powertrain efficiency, and control difficulty. It found that the dual-motor & power-separated type exhibits lower structural complexity than the other configurations, while both dual-motor configurations surpass the single-motor type in terms of powertrain efficiency. However, powertrain control is simpler in single-motor setups compared to dual-motor systems. Notably, the design requirements specified herein correspond to electric tractors with specific target specifications and may not universally apply to all electric tractors. Nonetheless, the methodology introduced can guide the determination of system requirements for electric tractors across varying usage environments.

Future research will expand on these findings by exploring dual-motor coupling powertrain configurations, particularly detailing the dual-motor & power-assist type, to compare and analyze powertrain performance further. Additionally, theoretical research will be conducted to compare powertrain performance against tractor operating points and power transmission efficiency to enhance the design of electric-tractor powertrains.

## Supplementary Information

Below is the link to the electronic supplementary material.

Supplementary Material 1

## Data Availability

The datasets used and/or analyzed during the current study available from the corresponding author on reasonable request.
