# Supplementary material for: Determination of design requirements and characteristic analysis of powertrain configurations for electric tractors based on actual agricultural workload
Source: Sci Rep. 2026 Mar 21;16:14381. doi: 10.1038/s41598-026-44453-0 (PMC13144337; doi:10.1038/s41598-026-44453-0)
Supplement: Supplementary file 1 — Supplementary Material 1 [file 41598_2026_44453_MOESM1_ESM.docx]

**Supplementary Material for “Determination of design requirements and characteristic analysis of powertrain configurations for electric tractors based on actual agricultural workload”**

Da-Vin Ahn ^a,b^, Ji-Tae Kim ^a,b^, Kyeongdae Kim ^a^, Gyu-Ha Han ^a^, Seung-Je Cho ^c^, Young-Jun Park ^a,b,d*^

**Supplemental Figure S1**

**Supplemental Figure S2**

**Supplemental Figure S3**

**Supplemental Figure S4**

**Supplemental Figure S5**

**Supplemental Figure S6**

|  |  |
| --- | --- |
| **(a) Tractor velocity** | **(b) Traction force** |
|  |  |
| **(c) Traction power** | **(d) Operation point of traction** |

Supplementary Fig. S1. Representative workload data during plow tillage with L3 gear.

|  |  |
| --- | --- |
| **(a) Tractor velocity** | **(b) Traction force** |
|  |  |
| **(c) Traction power** | **(d) Operation point of traction** |

Supplementary Fig. S2. Representative workload data during plow tillage with L4 gear.

|  |  |
| --- | --- |
| **(a) Tractor velocity** | **(b) Traction force** |
|  |  |
| **(c) Traction power** | **(d) Operation point of traction** |

Supplementary Fig. S3. Representative workload data during plow tillage with M2 gear.

|  |  |
| --- | --- |
| **(a) Tractor velocity** | **(b) Traction force** |
|  |  |
| **(c) Traction power** | **(d) Traction operating point** |
|  |  |
| **(e) PTO speed** | **(f) PTO torque** |
|  |  |
| **(g) PTO power** | **(h) PTO operating point** |

Supplementary Fig. S4. Representative workload total data during rotary tillage.

**Supplementary Fig. S5. Traction operating points overlaid on the traction requirement envelope.**

To address the concern regarding potential conservativeness, we quantified the margin in the force–speed domain as follows:

$$M_{F}=\frac{F_{env}\left( v \right)-F_{op}(v)}{F_{env}(v)}$$

Where, $M_{F}$ is margin of traction power envelope in operating point, $F_{env}\left( v \right)$ is traction force of traction power design requirement and $F_{op}(v)$ is traction force of agricultural workload.

Based on all measured operating points, the minimum margin was 0.063 % and the median margin was 89.83%, with 0 % of points exceeding the envelope.

**Supplementary Fig. S6. PTO operating points overlaid on the PTO requirement envelope**

The measured PTO operating points are overlaid with the PTO requirement envelope. We quantified the margin in the torque–speed domain as follows:

$$M_{T}=\frac{F_{env_{pto}}\left( \omega\right)-F_{op_{pto}}(\omega)}{{F_{env}}_{pto}(\omega)}$$

Where, $M_{T}$ is margin of PTO power envelope in operating point, $F_{env_{pto}}\left( \omega\right)$ is PTO torque of PTO power design requirement and $F_{op_{pto}}(\omega)$ is PTO torque of agricultural workload. Based on all measured operating points, the minimum margin was 0.001% and the median margin was 89.83 % with 0% of points exceeding the envelope.
